# Retrospective characterization of a rat model of volumetric muscle loss

**DOI:** 10.1186/s12891-022-05760-5

**Published:** 2022-08-26

**Authors:** Connor P. Dolan, Christopher L. Dearth, Benjamin T. Corona, Stephen M. Goldman

**Affiliations:** 1 DoD-VA Extremity Trauma and Amputation Center of Excellence, Bethesda, MD USA; 2 Department of Surgery, Uniformed Services University of the Health Sciences and Walter Reed National Military Medical Center, Bethesda, MD USA; 3 School of Medicine, Wake Forest University, Winston-Salem, NC USA

**Keywords:** Animal models, Trauma, Soft tissue injuries, Skeletal muscle, Pathophysiology

## Abstract

Volumetric muscle loss (VML) is a pervasive injury within contemporary combat and a primary driver of disability among injured Service members. As such, VML has been a topic of investigation over the past decade as the field has sought to understand the pathology of these injuries and to develop treatment strategies which restore the form and function of the involved musculature. To date, much of this work has been performed in disparate animal models that vary significantly in terms of the species utilized, the muscle (or muscle group) affected, and the volume of muscle lost. Moreover, variation exists in the reporting of anatomical and functional outcomes within these models. When taken together, the ability to successfully assess comparative efficacy of promising therapies is currently limited. As such, greater scrutiny on the characterization of these VML models is needed to better assess the quality of evidence supporting further translation of putative therapies. Thus, the objective of this study was to retrospectively characterize anatomical and functional outcomes associated with one such VML model – the 6 mm biopsy punch model of the rat tibialis anterior muscle. Through these efforts, it was shown that this model is highly reproducible and consistent across a large number of experiments. As such, the data presented herein represent a reasonable benchmark for the expected performance of this model with utility for drawing inferences across studies and identifying therapies which have shown promise within the preclinical domain, and thus are ready for further translation towards the clinic.

**Supplementary Information:**

The online version contains supplementary material available at 10.1186/s12891-022-05760-5.

## Introduction

Traumatic extremity injuries are highly reported in civilian populations [1] and are the most common survivable injuries experienced by US Service members since World War II [2]. Moreover, such injuries account for approximately two-thirds of initial hospital costs to the Military Health System [3]. Among such traumatic extremity injuries, volumetric muscle loss (VML) —operationally defined as the irrecoverable frank loss of skeletal muscle tissue [4] —is pervasive and a primary driver of disability among injured Service members due to its associated persistent functional deficits and lack of a standard of care [5]. As such, VML has been an unmet clinical need of increasing interest to the scientific and clinical communities for the last decade, and great efforts, and investments, have been made to uncover the pathophysiological underpinnings of this condition as well as to develop and evaluate a wide variety of treatment approaches, including those from the field of regenerative medicine.

To the latter point, a considerable portion of the existing literature on VML has been focused on developing regenerative therapies aimed at restoration of the form and function of the affected musculature. The efficacy of such therapies has largely been evaluated in several pre-clinical VML animal models; all of which aim to recapitulate aspects of the clinical condition but vary significantly in terms of the species utilized, the muscle (or muscle group) affected, and the volume of muscle lost [6]. While in some ways, diversity of models could be beneficial to facilitate a more comprehensive understanding of a clinically heterogeneous injury, however, it can also pose significant challenges to meaningful head-to-head comparisons between putative therapies which were evaluated in disparate models. As such, greater scrutiny on the characterization of these VML models is needed to better assess the quality of evidence supporting further translation of putative therapies. In order to accomplish that end state, one necessary step is to better understand the reproducibility and consistency of these animal models with respect to critical anatomical and functional outcomes so that the field, as a whole, can better evaluate the impact of novel therapies on the pathophysiology of VML injuries.

Perhaps the most commonly used VML injury model in recent years is a rat based unilateral injury model, in which a 6-mm biopsy punch is used to remove a full-thickness defect from the mid-belly of the tibialis anterior (TA) muscle with the contralateral hindlimb serving as an internal, uninjured control. To date, this model has been utilized for numerous studies (accounting for hundreds of animals) across multiple research groups and institutions (Table S1). The primary objective of this study was to retrospectively analyze a plurality of data associated with this VML model in an effort to present the important characteristics of the model including the reproducibility and consistency of critical outcome metrics. Secondarily, a comparison of this full thickness VML model to alternative partial thickness VML models affecting the TA muscle in rats was pursued to assess how readily results in varying models can be directly compared.

## Methods

### Animals and institutions

Studies utilizing the same unilateral, VML injury model based on a 6-mm full thickness biopsy punch of the TA muscle belly were identified within PubMed (Search strategy: “volumetric muscle loss” AND “rat” AND “tibialis anterior”) and corresponding authors were contacted requesting the subject level data for the untreated control animals reported in their manuscripts (Table S1). Additionally, corresponding authors were invited to provide unpublished controls from their laboratories for inclusion. Data from studies utilizing the same surgical procedure in a bilateral study design were excluded from the analysis. In total, data from 266 animals spread across 12 different experiments and 3 institutions was compiled and retrospectively analyzed. All protocols and animal care guidelines were approved by institutional animal care and use committees of their respective institutions. All experiments were conducted in compliance with the Animal Welfare Act, the Implementing Animal Welfare Regulations and in accordance with the principles of the Guide for the Care and Use of Laboratory Animals. Reporting is in accordance with ARRIVE 2.0 guidelines for reporting of in vivo experiments.

### Volumetric muscle loss injury model

**Fig. 1:**
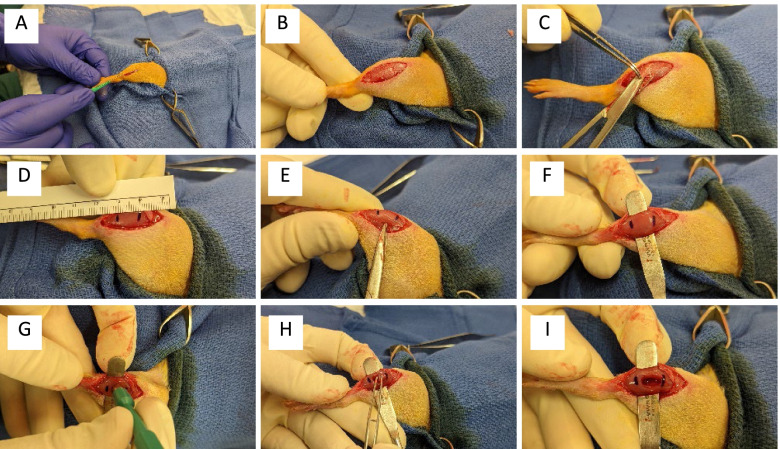
Volumetric muscle loss surgical procedure: an incision (**A**) is made on the lateral aspect of the rat’s hindlimb to reveal the underlying fascia (**B**). A small hole is made in the fascia with scissors (**C**) and the fascia is bluntly separated and reflected back from the underlying muscle. The middle third (**D**) of the TA muscle is demarcated and blunt scissors are used to penetrate the connective tissue between the TA and EDL muscles (**E**) to generate a tunnel beneath the TA. A spatula is inserted beneath the TA (**F**) and a 6 mm biopsy punch is used (**G**) to remove a full thickness section from the middle of the muscle belly (**H**) resulting in a VML defect (**I**)

All studies included in this retrospective analysis utilized the following standardized surgical approach for the generation of the unilateral VML injury in the TA muscle: (1) A lateral incision is made through the skin of the lower hindlimb, (2) the skin is separated from the fascia by blunt dissection, (3) the fascia is separated away from the muscle via sharp and blunt dissection, (4) skin and fascia are reflected from the anterior surface of the anterior crural muscles and (5) the middle third of the TA muscle is marked, (6) a metal plate is inserted between TA and extensor digitorum longus (EDL) muscles, and (7) a 6-mm punch biopsy is performed through the mid-belly of the TA muscle and is subsequently removed and weighed (Fig. 1). Any bleeding is controlled with light pressure, and the wound closed in layers with simple interrupted absorbable sutures.

### Muscle functional assessment and tissue collection

*In vivo* functional testing of TA muscles was performed in all studies investigated and collectively reported at 0, 3, 7, 14, 21, 28, 48, and 56-days post-injury. Briefly, TA muscle physiological properties were measured in anesthetized rats (isoflurane 1.5 – 2.0%) using dual-mode muscle lever systems (Aurora Scientific, Aurora, ON). Subcutaneous needle electrodes or implantable nerve cuffs were used to stimulate the common peroneal nerve. Optimal voltage was set with a series of tetanic contractions (150 Hz, 0.1 ms pulse width, 400 ms train). Then, a skin incision was made at the anterolateral aspect of the ankle and the distal tendon of the EDL was isolated and severed [7]. TA muscle isometric tetanic torque was measured (10–200 Hz) with the ankle at a right angle. In most studies, this procedure was then repeated on the contralateral, non-injured control limb. Isometric torque about the ankle is reported in units of N·mm according to each independently calibrated system. Footplate lengths were confirmed with each investigator for consistency in reporting. TA and EDL muscles were harvested from the injured and control limbs blotted dry and immediately weighed.

### Literature review & data extraction

A literature review was conducted to identify reports of alternative (partial thickness) VML models involving the TA muscle of rats to compare the consistency of outcomes associated with the neuromuscular function and gross anatomy of the TA muscle in the aforementioned full-thickness VML model. Twenty-four primary research articles published prior to March 1, 2021 of partial thickness VML models in the rat TA muscle were identified within PubMed (search strategy: “volumetric muscle loss” AND “rat” AND “tibialis anterior”). From the search results, reports were screened for the following inclusion criteria: (1) use of a partial thickness VML injuries involving the TA muscle, (2) reporting of endpoint body weights, (3) reporting of mass of tissue removed in the creation of the VML defect, and (4) reporting of tetanic isometric torque about the ankle, and (5) muscle wet weights for non-interventional control groups (negative control) as endpoint data, a minimum of 56 days postoperatively. Of the twenty-four search hits, 5 conforming reports were identified of which three reported multiple qualifying endpoints (Table S2). Values reported in units of force were transformed to corresponding torque units using the footplate length as the moment arm. Linear regressions of both endpoint tetanic isometric torque and TA wet weight, both normalized to body mass, were performed against defect wet weight for comparisons with the full-thickness biopsy punch VML model.

### Statistical analysis

Dependent variables were analyzed using analysis of variance (ANOVA) or paired t-tests. In the event of a significant ANOVA, a Fisher’s post-hoc test or a Sidak’s multiple comparison test was performed. Statistical significance was achieved at alpha of 0.05. Simple linear regression analyses were used to assess the relationships between skeletal muscle mass, body mass, and isometric torque production. For analyses involving the alternative partial thickness model, the regression model accounted for the sample size and standard deviation of the reported data. Differences in slopes of the linear regressions were determined by analysis of covariance. Data is presented as mean ± SEM. Sample sizes vary by experiment and endpoint according to primary data availability.

## Results

### Body weights and growth curves

**Fig. 2:**
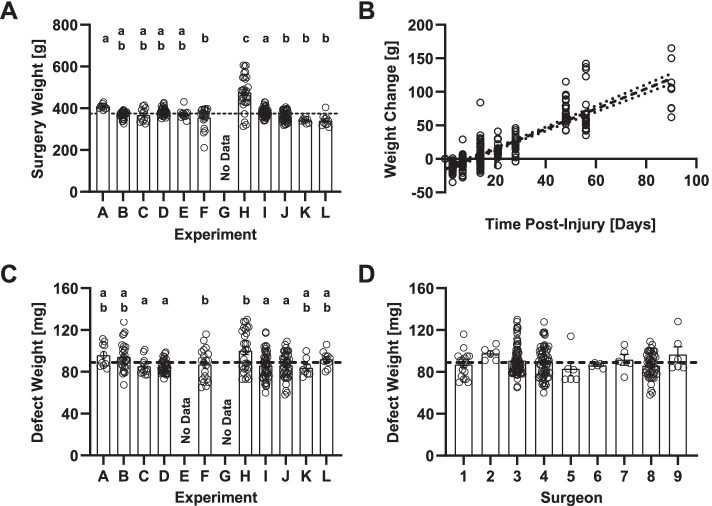
Body weight and surgical outcomes. **A** Animal weights at the time of surgery across 12 different experiments. The average animal weight across all experiments is indicated by the black-dashed line. Surgical body weight records were not available for Experiment G. **B** Animal weight change, calculated by subtracting the animal weight at study endpoints from the surgery weight, was found to decrease initially then increase linearly after 3 days post-operatively (*P* < 0.001). **C** Variation in defect weights across experiments. The average defect weight across all experiments is indicated by the black dashed line. Defect weights records were not available for experiment E or G. **D** Variation in defect weights by surgeon. The average defect weight across all experiments is indicated by the black dashed line. All panels represent individual data points as open circles. Bar graphs represent the group mean ± standard error. No difference between experimental groups (*P* > 0.05) was observed for groups marked with the same lower case letter annotation

The average weight of the animals prior to VML injury was 383 g ± 3 (*n* = 266) with slight variations across some of the experiments (Fig. 2A), and remain relatively unchanged for the first 21 days after injury. By 28 days post-VML, animals gained an average weight of 23.3 g ± 3.45 (*n* = 23, *P* = 0.006) relative to their weight at the time of VML injury, and continue growing in a linear fashion until the end of the experimental time course (Fig. 2B).

### VML injury creation

The average VML defect weight for all experiments (Fig. 2C) was 89.2 mg ± 0.9 (*n* = 266) and was found to vary across experiments (Main Effect, η^2^ = 0.15, *P* < 0.0001) with much of the variability explained by a single individual comparison (D vs. H, d = 1.061, *P* < 0.001). If defect weight is normalized to body weight the difference between the groups in this single comparison is diminished (D vs. H, d = 0.29, *P* = 0.260). Defect weight was not found to vary as a function of surgeon (Main Effect, η^2^ = 0.04, *P* < 0.001) (Fig. 2D).

### TA and EDL muscle weights

**Fig. 3:**
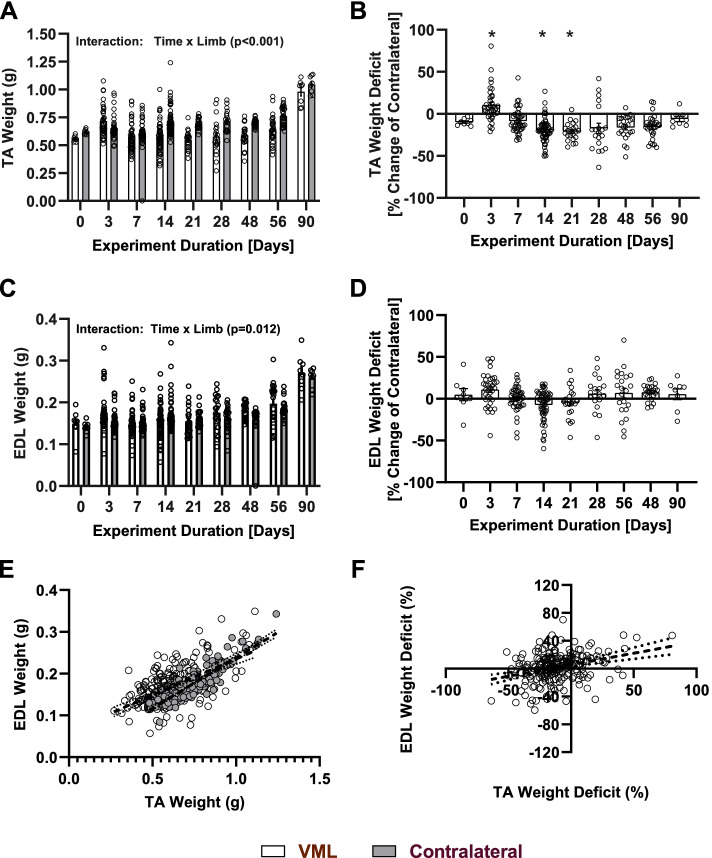
Muscle weight outcomes. Characteristic muscle weight dynamics are presented as absolute wet weights and as a percentage change of the VML hindlimb relative to its matched contralateral for study endpoints ranging from 3–90 days post-injury for both the tibialis anterior (TA) muscle (**A** and **B**) and the synergistic extensor longus (EDL) muscle (**C** and **D**), respectively. **E** Simple linear regression analysis comparing absolute weights of TA and EDL muscles in VML (clear) and healthy contralateral (grey) limbs with best fit lines and 95% confidence intervals represented by black and red dashed lines, respectively. **F** Simple linear regression of TA and EDL muscle weights presented as a percentage changes of the VML affected hindlimb relative to its matched contralateral. All panels represent data pooled from all experiments studied with individual data points represented as open circles. Bar graphs represent the group mean ± standard error. * Indicates a difference between VML and contralateral TA muscles at the specified timepoint (*P* < 0.05)

Changes in TA and EDL muscle (wet) weights at each experimental endpoint were investigated. When collapsed across all endpoints, the wet weights of VML injured TA muscles are reduced (Main Effect, η^2^ = 0.09, *P* < 0.001) relative to their matched contralateral limb (Fig. 3A). The primary exceptions to this observation are the 3-day and 7-day endpoints post-injury where wet weights are unchanged relative to their matched contralateral TA (Fig. 3B). However, VML-injured TA muscles weighed less than control TA muscles 14 days-post injury (*P* < 0.001) and remain as such for the remainder of experimental endpoints (Fig. 3B**)**. When investigating other synergistic muscles within the anterior compartment, a significant interaction between limb and time since injury was observed (Fig. 3C), although the percent difference between the matched muscle was never different from Day 0 for any of the endpoints studied (Fig. 3D). Simple linear regression analysis (Fig. 3E) demonstrated that TA and EDL weights were positively correlated in control limbs (Y = 0.242*X-0.004; *R*^2^ = 0.780). A positive correlation was also observed in VML-injured limbs (Y = 0.173*X + 0.062; *R*^2^ = 0.342), although the slopes of the correlations was flatter (*P* < 0.001). Subsequently, when analyzed between matched muscles within experimental animals, TA weight deficit and EDL weight deficit are likewise positively correlated (Y = 0.331*X + 5.555, *R*^2^ = 0.104) (Fig. 3F).

### *In-vivo* neuromuscular function

**Fig. 4:**
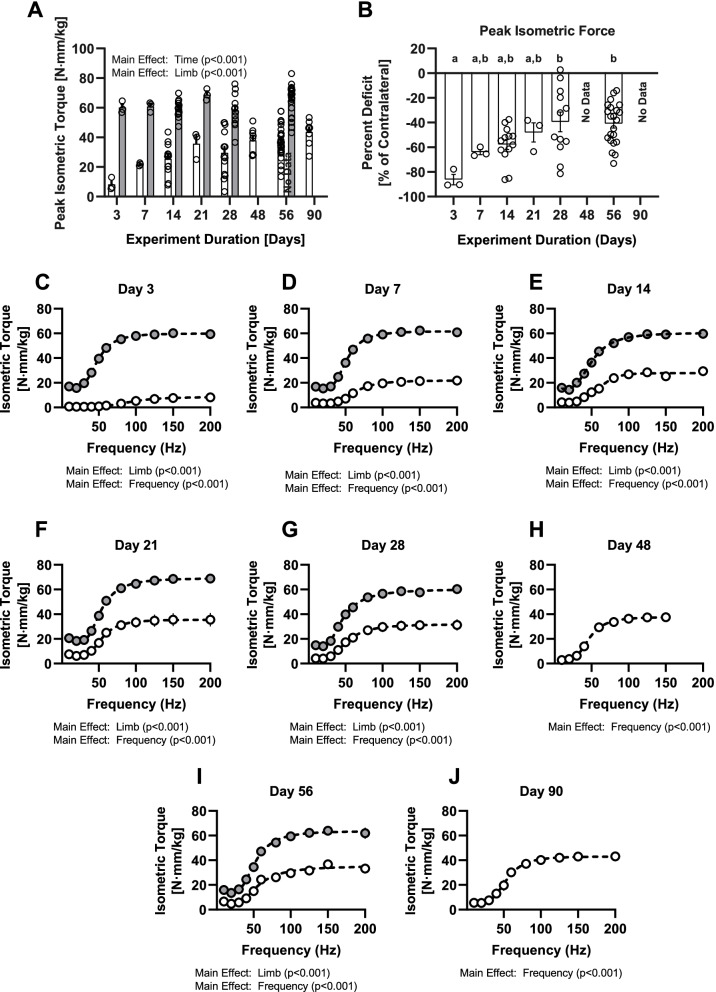
Functional outcomes. Peak isometric torque is presented normalized to endpoint body weight (**A**) and as a percentage change of the VML affected muscle (clear bar) to the matched, uninjured contralateral muscle.(grey bar) (**B**) for study endpoints ranging from 3–90 days post-injury. (**C**-**J**) Isometric torque frequency curves in VML affected TA muscles (clear) are compared with uninjured (grey) contralateral limbs at 3, 7, 14, 21, 28, 48, 90 and 90 days-post injury. All panels represent data pooled from all experiments studied with individual data points represented as open circles. Bar graphs represent the group mean ± standard error. No difference between experimental groups (*P* > 0.05) was observed for groups marked with the same lower case letter annotation

At all timepoints evaluated, there were no differences in peak isometric torque in the contralateral, control (i.e., uninjured) TA muscles (Fig. 4A). VML injured muscles exhibited a deficit in torque production about the ankle relative to unaffected contralateral control limbs at all time points (Fig. 4A, B). The deficit is most stark in the immediate post-acute time period as the peak isometric force of the VML-injured limbs was found to be decreased by -86% at 3-days post-VML injury. The magnitude of the functional deficit (relative to the uninjured control) decreased as a function of time (Main Effect, η^2^ = 0.32, *P* = 0.001), however, this recovery plateaued at 28 days post-injury as no differences were observed between timepoints after the 28-day timeframe (-39% vs. -41%, *P* > 0.999).

For all timepoints, and at all frequencies (10-200 Hz), the isometric torque from the VML-injured TA was less than control limbs (Fig. 4 C-J), but the torque-frequency curve characteristics, namely the hill slope, did not vary (η^2^ = 0.151, *P* = 0.215) across all of the timepoints investigated (Table S3). The frequency at which isometric torque reached its half-maximal value, however, was increased at 3 days post-VML injury relative to the uninjured contralateral limb (VML vs. Control: 89.4 ± 12.6 vs. 51.0 ± 9.9 Hz, *P* < 0.001). No such differences were observed between VML-injured and uninjured contralateral limbs for timepoints exceeding 7 days post-injury (*P* > 0.05), although there is a statistically significant linear trend (*P* < 0.001) towards lower half-maximal frequencies with time since injury.

### Comparison with partial thickness VML models

**Fig. 5:**
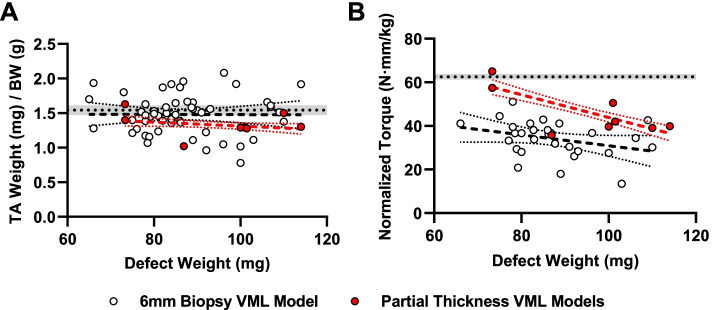
Comparison of outcomes with partial thickness VML models. Simple linear regressions with 95% confidence intervals are presented for (**A**) tibialis anterior wet weight and (**B**) body weight normalized peak isometric torque as a function of surgical defect weight for the 6 mm biopsy VML model (clear) and published reports of alternative partial thickness VML models (red) at chronic timepoints (> 48 days post-injury). Values representing the mean ± SEM of the healthy contralateral limbs from the 6 mm biopsy data points are presented by the horizontal dashed line and associated shading

Normalized TA wet weights for the full-thickness punch biopsy VML model were higher (*P* < 0.05) than those of the partial thickness VML models identified (Fig. 5A). Neither data set, however, showed a meaningful relationship between normalized TA wet weight at chronic timepoints (> 48 day post-operatively) as a function of the amount of tissue removed (i.e., defect wet weight). With respect to end organ functional output, the dependency of normalized peak isometric torque at the longest endpoints on surgical defect weight, was different (*P* < 0.05) between the data sets (Fig. 5B). The full-thickness punch biopsy VML model studied herein was less susceptible to variations in surgical defect wet (Y = -0.2490X + 55.82, *R*^2^ = 0.10) relative to the collection of partial thickness VML models (Y = -0.521X + 96.02, *R*^2^ = 0.49).

## Discussion

The retrospective analysis performed herein illustrates several important characteristics of the rat, biopsy punch, TA-based VML model. Most importantly, this analysis has shown that this VML injury model is both acceptably reproducible with respect to surgical technique and consistent across studies with respect to key experimental outcomes of interest, namely muscle weights and torque production. Moreover, this model has been leveraged to understand much about the pathobiological underpinnings of VML, including (but not limited to): alterations in a dysregulated immune-inflammatory response [8], alterations to muscle architecture [9], axotomy of motoneurons [10], and secondary denervation and destabilization of neuromuscular junctions [11]. As such, it has become the workhorse model in numerous laboratories and a prime candidate for adoption by investigators entering the field to use as testbed for the evaluation of putative therapies for continued translational development.

In addition to demonstrating the consistency of this model, this retrospective analysis has generated a large data set that illuminates more subtle nuances of the model than is typically observed in single prospective studies. First, with respect to muscle weight, TA muscles affected by VML were shown to consistently have reduced wet weights relative to their matched, unaffected contralateral limbs for experimental endpoints greater than 1 weeks post-injury. However, at 3- and 7-days post-injury, wet weights are similar between injured and uninjured contralateral muscles (Fig. 3A), even though with an average of 88.9 ± 1.1 mg (Fig. 2C) of muscle was removed days earlier. This observation is likely explained by early inflammation and edema in the initial days following the VML injury and suggests that one should see the bulk of this effect recede within two weeks (Fig. 3A). A second interesting observation is that EDL weights from the ipsilateral and contralateral limbs do not differ from each other at any time point. This observation is important for two reasons. First, it illustrates that compartmental muscle atrophy due to neural injury proximal to the TA muscle is not present and is not a primary driving factor of the observed pathology. This is an important realization as common peroneal nerve damage proximal to the TA muscle could confound results, and halt development of an otherwise promising therapy. Second, the EDL muscle is a synergist to the TA, and thus one would expect the masses of the two muscles would be highly correlated under homeostatic conditions (Fig. 3E**)**. Such a relationship, however, is not manifested between the TA and EDL muscle in this model (Fig. 3, Panels E–F) as the slope of the relationship is considerably flatter in the VML affected limb relative to the muscles of the unaffected contralateral. Prior studies interrogating whether surgical ablation of the EDL results in TA overloading showed a similar lack of compensation between these two synergist muscles [12]. A lack of observed hypertrophy could plausibly be explained by alterations in gait over time which compensate for the presence of the VML injury by altering the demands on the anterior crural muscles, a strategy that has been illustrated in VML models of a different geometry affecting the TA muscle in recent reports [13].

When compared with a collection of partial-thickness VML models involving the rat TA muscle, we find that chronic, neural-evoked muscle function of the full-thickness VML model studies herein was less influenced by initial injury creation (i.e., VML defect weight). While reduced variation in outcomes of the model owing to surgical manipulation is a strong indicator of the consistency of our model, this finding also highlights the need for further investigation into other topics such as the extent to which the geometry of the VML defect contributes to overall myofiber damage, denervation, vascular injury, and contractile force transmission. Specifically with respect to myofiber damage, the comparison between partial and full thickness injuries studied herein suggests the total number of myofibers injured and extent of myofiber injury may be of greater importance to functional outcomes of VML models than previously appreciated. In other words, if total defect mass is held constant, one would expect that a full-thickness injury would ablate a lower proportion of total sarcomeres within a given myofiber relative to a partial thickness defect. This is not to say that one injury pattern is better than another, but rather that subtle differences in models could potentially influence the interpretation of results for any study investigating a particular intervention.

One factor that was not thoroughly investigated in this study was the impact of sex differences on study outcomes in the model. The primary reason this factor was not studied in this analysis is that the overwhelming majority of studies using the 6 mm biopsy punch model have focused solely on male rats. Historically, this has been the case as these studies were performed at military research organizations intent on serving the needs of combat wounded Service members of the U.S. Military, a population that is overwhelmingly male. As the field expands, however, the need to investigate the impact of sex difference on outcomes of VML is of great importance given the role testosterone impacts skeletal muscle growth and regeneration. Such studies will be necessary to ensure findings are most broadly impactful across all military and civilian trauma populations.

While the primary intent of this retrospective analysis was to determine the reproducibility and consistency of the 6 mm biopsy punch VML model in the TA muscle, the size and robustness of the data set generated does begin to raise ethical and economic arguments for augmenting the standard experimental approaches to evaluation of therapies within the preclinical domain. While in keeping with good experimental design, it is standard to include a control group/cohort that are injured but do not receive an intervention (i.e., a negative control); however, in light of the data presented herein, one could reasonably argue for reducing the number of negative control animals on a per study basis to a minimal essential number to confirm the absence of experimental drift from the historical norms presented herein. While we are not advocating for complete omission of negative control groups from future studies, we do hope that the data set generated herein might be built upon to work towards establishing a bona fide normative data set that could be judiciously used as a reference for the expected outcome in lieu of additional study animals in instances where their use is appropriate to the intent of the scientific inquiry.

## Conclusion

This manuscript represents an important resource for investigators within the field of orthopedic trauma, namely those investigating VML injuries, who may seek to use a robust injury model for the evaluation of novel therapies developed in their laboratories. The data presented herein thoroughly establish benchmarks for the expected performance of the model with respect to common primary outcome measures. Such benchmarks are important for assessing the quality of research data generated within a particular study as they enhance the ability of the field to compare against historical norms. In doing so, it better enables the clinical and scientific communities to make reasonable inferences on the comparative effectiveness of promising therapies across the literature. Moreover, proper scientific usage of such benchmarks may enable more judicious use of laboratory animals, increased efficiency, and more rapid translation of promising therapies upwards towards the clinic.

## Supplementary Information

**Additional file 1. **Supplemental data: raw data repository.

**Additional file 2: Table S1.** Studies utilizing the 6 mm biopsy punch model of VML in the rat tibialis anterior muscle [9, 11, 14–27]. **Table S2.** Published studies included for comparative analysis using partial thickness models of VML in the rat TA muscle. **Table S3.** Best fit parameters of sigmoidal fit for torque-frequency relationship of TA muscles with VML.

## Data Availability

All data generated or analyzed during this study are included in this published article (and its supplementary information files).
