# Supplementary material for: Retrospective characterization of a rat model of volumetric muscle loss
Source: BMC Musculoskelet Disord. 2022 Aug 26;23:814. doi: 10.1186/s12891-022-05760-5 (PMC9414143; doi:10.1186/s12891-022-05760-5)
Supplement: Supplementary file 1 — Additional file 1. Supplemental data: raw data repository. [file 12891_2022_5760_MOESM1_ESM.docx]

| Experiment Letter | Location | Animal ID | Surgeon | Duration | Defect  Weight  [mg] | Sx. Weight [g] | Endpoint Weight  [g] | Left  TA [g] | Right  TA [g] | Left EDL [g] | Right EDL [g] | Unaffected Limb: Torque (N*mm) | | | | | | | | | | | Affected Limb: Torque (N*mm) | | | | | | | | | | |
| --- | --- | --- | --- | --- | --- | --- | --- | --- | --- | --- | --- | --- | --- | --- | --- | --- | --- | --- | --- | --- | --- | --- | --- | --- | --- | --- | --- | --- | --- | --- | --- | --- | --- |
|  |  |  |  |  |  |  |  |  |  |  |  | 10 | 20 | 30 | 40 | 50 | 60 | 80 | 100 | 125 | 150 | 200 | 10 | 20 | 30 | 40 | 50 | 60 | 80 | 100 | 125 | 150 | 200 |
| A | ISR | EXPA.001 | 3 | 14 DAYS | 111.6 | 422 | 411 | 0.66 | 0.72 | 0.14 | 0.17 | - | - | - | - | - | - | - | - | - | - | - | - | - | - | - | - | - | - | - | - | - | - |
| A | ISR | EXPA.002 | 3 | 14 DAYS | 105.2 | 408 | 408 | 0.59 | 0.74 | 0.17 | 0.17 | - | - | - | - | - | - | - | - | - | - | - | - | - | - | - | - | - | - | - | - | - | - |
| A | ISR | EXPA.003 | 6 | 14 DAYS | 88.6 | 400 | 408 | 0.60 | 0.74 | 0.18 | 0.17 | - | - | - | - | - | - | - | - | - | - | - | - | - | - | - | - | - | - | - | - | - | - |
| A | ISR | EXPA.004 | 6 | 14 DAYS | 85.4 | 400 | 391 | 0.62 | 0.71 | 0.13 | 0.18 | - | - | - | - | - | - | - | - | - | - | - | - | - | - | - | - | - | - | - | - | - | - |
| A | ISR | EXPA.005 | 3 | 56 DAYS | 96.2 | 407 | 450 | 0.94 | 0.83 | 0.16 | 0.18 | 7.90 | 6.85 | 9.03 | 13.28 | 20.38 | 26.89 | 32.52 | 34.01 | 35.19 | 36.23 | 36.73 | 13.23 | 11.87 | 15.87 | 25.63 | 40.63 | 56.12 | 71.79 | 77.04 | 80.38 | 82.28 | 83.09 |
| A | ISR | EXPA.006 | 3 | 56 DAYS | 109 | 410 | 463 | 0.70 | 0.80 | 0.23 | 0.18 | 9.69 | 9.20 | 13.68 | 21.71 | 29.87 | 34.21 | 38.29 | 40.33 | 41.61 | 42.29 | 42.63 | 15.09 | 13.71 | 18.59 | 26.85 | 40.14 | 51.53 | 61.21 | 65.01 | 67.14 | 68.29 | 68.74 |
| A | ISR | EXPA.007 | 3 | 56 DAYS | 106.2 | 390 | 450 | 0.75 | 0.91 | 0.22 | 0.23 | 3.91 | 3.37 | 4.36 | 7.28 | 12.39 | 19.59 | 28.54 | 31.52 | 33.38 | 34.25 | 34.46 | 18.08 | 16.23 | 21.02 | 33.69 | 51.40 | 62.49 | 70.28 | 72.97 | 74.86 | 75.97 | 74.79 |
| A | ISR | EXPA.008 | 6 | 56 DAYS | 86.2 | 430 | 475 | 0.65 | 0.84 | 0.24 | 0.19 | 6.75 | 5.98 | 7.84 | 12.41 | 20.15 | 26.75 | 32.27 | 34.18 | 35.42 | 36.05 | 36.26 | 19.60 | 17.84 | 24.53 | 37.70 | 50.85 | 59.24 | 66.21 | 69.18 | 71.02 | 71.90 | 71.22 |
| A | ISR | EXPA.009 | 6 | 56 DAYS | 82.9 | 410 | 460 | 0.69 | 0.82 | 0.23 | 0.24 | 6.49 | 5.69 | 7.26 | 12.53 | 20.81 | 26.20 | 30.32 | 32.15 | 33.25 | 33.75 | 33.84 | 18.96 | 17.57 | 23.68 | 37.45 | 51.90 | 59.30 | 65.43 | 68.26 | 70.16 | 71.07 | 69.84 |
| A | ISR | EXPA.010 | 6 | 56 DAYS | 87.7 | 397 | 440 | 0.73 | 0.85 | 0.23 | 0.18 | 5.15 | 4.39 | 5.47 | 8.85 | 14.60 | 21.81 | 28.45 | 30.69 | 31.73 | 31.86 | 31.84 | - | - | - | - | - | - | - | - | - | - | - |
| B | ISR | EXPB.001 | 3 | 03 DAYS | 79.1 | 349 | 344 | 0.87 | 0.63 | 0.16 | 0.16 | 0.57 | 0.54 | 0.49 | 0.53 | 0.69 | 0.90 | 1.94 | 3.29 | 5.05 | 5.61 | 6.26 | - | 18.85 | 23.01 | 30.76 | 42.00 | 50.94 | 58.04 | 61.21 | 63.42 | 64.62 | 64.66 |
| B | ISR | EXPB.002 | 3 | 03 DAYS | 91.2 | 326 | 318 | 0.75 | 0.49 | 0.17 | 0.12 | 1.30 | 1.01 | 1.04 | 1.08 | 1.22 | 2.00 | 3.67 | 7.97 | 10.52 | 12.27 | 13.07 | 14.81 | 14.51 | 19.94 | 29.66 | 40.96 | 50.41 | 57.00 | 58.80 | 58.27 | 58.98 | 57.93 |
| B | ISR | EXPB.003 | 3 | 03 DAYS | 84.1 | 345 | 339 | 0.51 | 0.50 | 0.18 | 0.13 | 0.37 | 0.38 | 0.32 | 0.34 | 0.57 | 1.61 | 3.73 | 4.59 | 5.05 | 5.30 | 5.34 | 15.62 | 14.20 | 16.23 | 24.30 | 35.45 | 43.29 | 50.67 | 53.67 | 55.85 | 56.85 | 55.80 |
| B | ISR | EXPB.004 | 4 | 03 DAYS | 101.6 | 363 | 351 | 0.69 | 0.65 | 0.15 | 0.18 | - | - | - | - | - | - | - | - | - | - | - | - | - | - | - | - | - | - | - | - | - | - |
| B | ISR | EXPB.005 | 4 | 03 DAYS | 127.8 | 370 | 360 | 0.54 | 0.64 | 0.20 | 0.13 | - | - | - | - | - | - | - | - | - | - | - | - | - | - | - | - | - | - | - | - | - | - |
| B | ISR | EXPB.006 | 4 | 03 DAYS | 92.1 | 382 | 369 | 0.56 | 0.70 | 0.16 | 0.14 | - | - | - | - | - | - | - | - | - | - | - | - | - | - | - | - | - | - | - | - | - | - |
| B | ISR | EXPB.007 | 4 | 03 DAYS | 104.5 | 377 | 360 | 0.85 | 0.73 | 0.17 | 0.14 | - | - | - | - | - | - | - | - | - | - | - | - | - | - | - | - | - | - | - | - | - | - |
| B | ISR | EXPB.008 | 4 | 03 DAYS | 100.7 | 369 | 362 | 0.66 | 0.66 | 0.16 | 0.14 | - | - | - | - | - | - | - | - | - | - | - | - | - | - | - | - | - | - | - | - | - | - |
| B | ISR | EXPB.009 | 3 | 07 DAYS | 79.7 | 346 | 350 | 0.46 | 0.57 | 0.14 | 0.15 | 4.06 | 3.60 | 4.01 | 5.50 | 7.68 | 12.35 | 17.00 | 19.12 | 19.93 | 20.49 | 20.79 | 18.98 | 17.18 | 18.14 | 24.59 | 35.34 | 46.10 | 55.33 | 58.63 | 60.61 | 61.70 | 62.41 |
| B | ISR | EXPB.010 | 3 | 07 DAYS | 90.2 | 338 | 345 | 0.46 | 0.58 | 0.18 | 0.16 | 3.92 | 3.38 | 3.43 | 5.30 | 8.50 | 12.43 | 17.74 | 20.12 | 21.51 | 22.32 | 22.87 | - | - | - | - | - | - | - | - | - | - | 56.96 |
| B | ISR | EXPB.011 | 3 | 07 DAYS | 85 | 337 | 333 | 0.50 | 0.52 | 0.16 | 0.15 | 3.58 | 2.91 | 2.79 | 3.61 | 5.29 | 9.69 | 17.07 | 19.55 | 20.79 | 21.43 | 21.88 | 14.88 | 13.54 | 15.75 | 24.79 | 37.09 | 47.57 | 56.18 | 59.67 | 61.74 | 63.03 | 63.29 |
| B | ISR | EXPB.012 | 4 | 07 DAYS | 117.9 | 373 | 370 | 0.58 | 0.68 | 0.10 | 0.16 | - | - | - | - | - | - | - | - | - | - | - | - | - | - | - | - | - | - | - | - | - | - |
| B | ISR | EXPB.013 | 4 | 07 DAYS | 67.5 | 358 | 333 | 0.48 | 0.58 | 0.13 | 0.12 | - | - | - | - | - | - | - | - | - | - | - | - | - | - | - | - | - | - | - | - | - | - |
| B | ISR | EXPB.014 | 4 | 07 DAYS | 80.6 | 383 | 387 | 0.73 | 0.72 | 0.18 | 0.15 | - | - | - | - | - | - | - | - | - | - | - | - | - | - | - | - | - | - | - | - | - | - |
| B | ISR | EXPB.015 | 4 | 07 DAYS | 87 | 369 | 364 | 0.59 | 0.61 | 0.14 | 0.14 | - | - | - | - | - | - | - | - | - | - | - | - | - | - | - | - | - | - | - | - | - | - |
| B | ISR | EXPB.016 | 3 | 14 DAYS | 89.5 | 382 | 384 | 0.55 | 0.64 | - | - | 7.06 | 6.42 | 7.39 | 9.87 | 14.12 | 23.40 | 36.18 | 40.15 | 42.09 | 43.13 | 43.59 | 15.53 | 14.52 | 17.99 | 26.71 | 38.92 | 51.30 | 61.39 | 65.24 | 67.83 | 69.15 | 69.83 |
| B | ISR | EXPB.017 | 3 | 14 DAYS | 93.7 | 392 | 378 | 0.69 | 0.66 | - | - | 5.82 | 4.79 | 5.09 | 7.22 | 10.85 | 18.68 | 28.36 | 31.67 | 33.67 | 34.67 | 35.12 | 19.92 | 18.26 | 19.81 | 25.47 | 34.46 | 46.57 | 56.69 | 60.62 | 63.30 | 64.42 | 62.22 |
| B | ISR | EXPB.018 | 3 | 14 DAYS | 85.8 | 381 | 376 | 0.49 | 0.61 | - | - | 3.50 | 2.92 | 3.22 | 4.44 | 7.09 | 12.31 | 22.26 | 25.24 | 26.93 | 27.87 | 28.54 | 15.67 | 13.34 | 14.18 | 19.73 | 29.23 | 42.31 | 54.59 | 58.65 | 61.11 | 62.36 | 63.02 |
| B | ISR | EXPB.019 | 4 | 14 DAYS | 90.5 | 364 | 360 | 0.48 | 0.66 | 0.16 | 0.16 | - | - | - | - | - | - | - | - | - | - | - | - | - | - | - | - | - | - | - | - | - | - |
| B | ISR | EXPB.020 | 4 | 14 DAYS | 96 | 377 | 370 | 0.59 | 0.78 | 0.16 | 0.16 | - | - | - | - | - | - | - | - | - | - | - | - | - | - | - | - | - | - | - | - | - | - |
| B | ISR | EXPB.021 | 4 | 14 DAYS | 103.7 | 362 | 372 | 0.66 | 0.68 | 0.16 | 0.17 | - | - | - | - | - | - | - | - | - | - | - | - | - | - | - | - | - | - | - | - | - | - |
| B | ISR | EXPB.022 | 4 | 14 DAYS | 90.9 | 377 | 375 | 0.56 | 0.63 | 0.15 | 0.14 | - | - | - | - | - | - | - | - | - | - | - | - | - | - | - | - | - | - | - | - | - | - |
| B | ISR | EXPB.023 | 3 | 21 DAYS | 78.1 | 388 | 400 | 0.49 | 0.70 | 0.13 | 0.17 | 5.17 | 4.16 | 5.16 | 8.78 | 15.54 | 20.84 | 23.45 | 24.51 | 24.81 | 25.05 | 24.53 | 22.50 | 20.25 | 21.44 | 29.87 | 42.76 | 53.52 | 61.48 | 64.71 | 66.92 | 68.25 | 68.57 |
| B | ISR | EXPB.024 | 3 | 21 DAYS | 88.6 | 379 | 369 | 0.48 | 0.66 | 0.11 | 0.21 | 8.53 | 7.36 | 8.64 | 11.90 | 18.04 | 27.35 | 34.38 | 37.02 | 38.95 | 39.88 | 40.27 | 19.08 | 17.14 | 18.04 | 25.74 | 37.80 | 48.94 | 57.66 | 60.90 | 63.54 | 64.91 | 65.21 |
| B | ISR | EXPB.025 | 3 | 21 DAYS | 98 | 382 | 395 | 0.57 | 0.71 | 0.24 | 0.18 | 8.99 | 7.17 | 7.14 | 10.14 | 16.72 | 26.88 | 35.59 | 38.59 | 40.47 | 41.58 | 41.94 | 20.22 | 17.55 | 17.94 | 23.73 | 35.71 | 50.22 | 63.70 | 68.38 | 71.25 | 72.52 | 72.78 |
| B | ISR | EXPB.026 | 4 | 21 DAYS | 115.2 | 381 | 395 | 0.52 | 0.62 | 0.14 | 0.15 | - | - | - | - | - | - | - | - | - | - | - | - | - | - | - | - | - | - | - | - | - | - |
| B | ISR | EXPB.027 | 4 | 21 DAYS | 105.7 | 390 | 412 | 0.46 | 0.76 | 0.18 | 0.19 | - | - | - | - | - | - | - | - | - | - | - | - | - | - | - | - | - | - | - | - | - | - |
| B | ISR | EXPB.028 | 4 | 21 DAYS | 116.2 | 372 | 401 | 0.58 | 0.78 | 0.12 | 0.16 | - | - | - | - | - | - | - | - | - | - | - | - | - | - | - | - | - | - | - | - | - | - |
| C | ISR | EXPC.001 | 3 | 14 DAYS | 81.1 | 337 | 340 | 0.48 | 0.61 | 0.12 | 0.14 | - | - | - | - | - | - | - | - | - | - | - | - | - | - | - | - | - | - | - | - | - | - |
| C | ISR | EXPC.002 | 3 | 14 DAYS | 99.2 | 340 | 360 | 0.36 | 0.64 | 0.09 | 0.17 | - | - | - | - | - | - | - | - | - | - | - | - | - | - | - | - | - | - | - | - | - | - |
| C | ISR | EXPC.003 | 3 | 14 DAYS | 101.3 | 334 | 344 | 0.53 | 0.58 | 0.12 | 0.14 | - | - | - | - | - | - | - | - | - | - | - | - | - | - | - | - | - | - | - | - | - | - |
| C | ISR | EXPC.004 | 3 | 14 DAYS | 90.5 | 345 | 347 | 0.42 | 0.62 | 0.13 | 0.15 | - | - | - | - | - | - | - | - | - | - | - | - | - | - | - | - | - | - | - | - | - | - |
| C | ISR | EXPC.005 | 3 | 14 DAYS | 80.7 | 326 | 349 | 0.47 | 0.60 | 0.12 | 0.14 | - | - | - | - | - | - | - | - | - | - | - | - | - | - | - | - | - | - | - | - | - | - |
| C | ISR | EXPC.006 | 3 | 14 DAYS | 77.3 | 350 | 351 | 0.32 | 0.53 | 0.10 | 0.15 | - | - | - | - | - | - | - | - | - | - | - | - | - | - | - | - | - | - | - | - | - | - |
| C | ISR | EXPC.007 | 3 | 56 DAYS | 86.8 | 401 | 459 | 0.90 | 0.79 | 0.23 | 0.18 | 5.56 | 4.35 | 3.86 | 5.83 | 8.99 | 14.45 | 31.38 | 34.23 | 36.01 | 36.58 | 36.75 | - | - | - | - | - | - | - | - | - | - | - |
| C | ISR | EXPC.008 | 3 | 56 DAYS | 79.2 | 392 | 442 | 0.64 | 0.85 | 0.25 | 0.18 | 2.69 | 2.41 | 3.49 | 6.83 | 12.75 | 16.38 | 19.17 | 20.19 | 20.61 | 20.84 | 20.54 | - | - | - | - | - | - | - | - | - | - | - |
| C | ISR | EXPC.009 | 3 | 56 DAYS | 78.9 | 411 | 453 | 0.52 | 0.85 | 0.21 | 0.18 | 4.83 | 4.29 | 5.03 | 7.64 | 13.27 | 19.32 | 24.83 | 27.41 | 28.82 | 29.13 | 29.39 | - | - | - | - | - | - | - | - | - | - | - |
| C | ISR | EXPC.010 | 3 | 56 DAYS | 77.1 | 379 | 414 | 0.63 | 0.75 | 0.14 | 0.20 | 5.09 | 4.37 | 5.59 | 9.22 | 15.90 | 22.91 | 28.56 | 30.99 | 32.27 | 33.00 | 33.25 | - | - | - | - | - | - | - | - | - | - | - |
| C | ISR | EXPC.011 | 3 | 56 DAYS | 82.8 | 385 | 440 | 0.70 | 0.74 | 0.18 | 0.17 | 8.11 | 7.04 | 8.47 | 13.36 | 20.43 | 26.83 | 32.13 | 34.72 | 36.69 | 37.62 | 38.11 | - | - | - | - | - | - | - | - | - | - | - |
| C | ISR | EXPC.012 | 3 | 56 DAYS | 88.7 | 415 | 472 | 0.75 | 0.88 | 0.24 | 0.18 | 5.00 | 5.16 | 6.53 | 10.33 | 16.13 | 25.23 | 34.21 | 38.13 | 39.87 | 40.94 | 41.12 | - | - | - | - | - | - | - | - | - | - | - |
| D | ISR | EXPD.001 | 3 | 03 DAYS | 88.4 | 375 | 357 | 1.00 | 0.55 | 0.19 | 0.13 | - | - | - | - | - | - | - | - | - | - | - | - | - | - | - | - | - | - | - | - | - | - |
| D | ISR | EXPD.002 | 3 | 03 DAYS | 89.7 | 364 | 352 | 0.72 | 0.66 | 0.17 | 0.14 | - | - | - | - | - | - | - | - | - | - | - | - | - | - | - | - | - | - | - | - | - | - |
| D | ISR | EXPD.003 | 3 | 03 DAYS | 73.3 | 367 | 361 | 0.73 | 0.61 | 0.08 | 0.14 | - | - | - | - | - | - | - | - | - | - | - | - | - | - | - | - | - | - | - | - | - | - |
| D | ISR | EXPD.004 | 3 | 03 DAYS | 86.3 | 379 | 372 | 0.73 | 0.56 | 0.18 | 0.14 | - | - | - | - | - | - | - | - | - | - | - | - | - | - | - | - | - | - | - | - | - | - |
| D | ISR | EXPD.005 | 3 | 03 DAYS | 79.1 | 400 | 380 | 0.74 | 0.61 | 0.17 | 0.14 | - | - | - | - | - | - | - | - | - | - | - | - | - | - | - | - | - | - | - | - | - | - |
| D | ISR | EXPD.006 | 3 | 03 DAYS | 90.5 | 421 | 397 | 0.67 | 0.64 | 0.18 | 0.15 | - | - | - | - | - | - | - | - | - | - | - | - | - | - | - | - | - | - | - | - | - | - |
| D | ISR | EXPD.007 | 3 | 03 DAYS | 90.6 | 406 | 392 | 0.64 | 0.60 | 0.17 | 0.13 | - | - | - | - | - | - | - | - | - | - | - | - | - | - | - | - | - | - | - | - | - | - |
| D | ISR | EXPD.008 | 3 | 03 DAYS | 83.2 | 411 | 396 | 0.63 | 0.61 | 0.17 | 0.15 | - | - | - | - | - | - | - | - | - | - | - | - | - | - | - | - | - | - | - | - | - | - |
| D | ISR | EXPD.009 | 3 | 07 DAYS | 77.8 | 354 | 345 | 0.89 | 0.63 | 0.21 | 0.16 | - | - | - | - | - | - | - | - | - | - | - | - | - | - | - | - | - | - | - | - | - | - |
| D | ISR | EXPD.010 | 3 | 07 DAYS | 80.5 | 362 | 351 | 0.69 | 0.63 | 0.16 | 0.14 | - | - | - | - | - | - | - | - | - | - | - | - | - | - | - | - | - | - | - | - | - | - |
| D | ISR | EXPD.011 | 3 | 07 DAYS | 85.6 | 362 | 364 | 0.78 | 0.68 | 0.13 | 0.14 | - | - | - | - | - | - | - | - | - | - | - | - | - | - | - | - | - | - | - | - | - | - |
| D | ISR | EXPD.012 | 3 | 07 DAYS | 82.4 | 371 | 371 | 0.51 | 0.59 | 0.15 | 0.15 | - | - | - | - | - | - | - | - | - | - | - | - | - | - | - | - | - | - | - | - | - | - |
| D | ISR | EXPD.013 | 3 | 07 DAYS | 84.3 | 376 | 366 | 0.58 | 0.59 | 0.14 | 0.13 | - | - | - | - | - | - | - | - | - | - | - | - | - | - | - | - | - | - | - | - | - | - |
| D | ISR | EXPD.014 | 3 | 07 DAYS | 78.5 | 382 | 361 | 0.58 | 0.60 | 0.15 | 0.14 | - | - | - | - | - | - | - | - | - | - | - | - | - | - | - | - | - | - | - | - | - | - |
| D | ISR | EXPD.015 | 3 | 07 DAYS | 79.4 | 382 | 382 | 0.54 | 0.62 | 0.14 | 0.14 | - | - | - | - | - | - | - | - | - | - | - | - | - | - | - | - | - | - | - | - | - | - |
| D | ISR | EXPD.016 | 3 | 07 DAYS | 92.5 | 392 | 392 | 0.47 | 0.58 | 0.14 | 0.14 | - | - | - | - | - | - | - | - | - | - | - | - | - | - | - | - | - | - | - | - | - | - |
| D | ISR | EXPD.017 | 3 | 14 DAYS | 87.9 | 400 | 385 | 0.82 | 0.64 | 0.17 | 0.16 | - | - | - | - | - | - | - | - | - | - | - | - | - | - | - | - | - | - | - | - | - | - |
| D | ISR | EXPD.018 | 3 | 14 DAYS | 81.6 | 387 | 382 | 0.74 | 0.65 | 0.09 | 0.13 | - | - | - | - | - | - | - | - | - | - | - | - | - | - | - | - | - | - | - | - | - | - |
| D | ISR | EXPD.019 | 3 | 14 DAYS | 97.7 | 394 | 392 | 0.66 | 0.64 | 0.15 | 0.14 | - | - | - | - | - | - | - | - | - | - | - | - | - | - | - | - | - | - | - | - | - | - |
| D | ISR | EXPD.020 | 3 | 14 DAYS | 82.5 | 360 | 339 | 0.61 | 0.61 | 0.15 | 0.14 | - | - | - | - | - | - | - | - | - | - | - | - | - | - | - | - | - | - | - | - | - | - |
| D | ISR | EXPD.021 | 3 | 14 DAYS | 94.5 | 381 | 378 | 0.61 | 0.70 | 0.15 | 0.14 | - | - | - | - | - | - | - | - | - | - | - | - | - | - | - | - | - | - | - | - | - | - |
| D | ISR | EXPD.022 | 3 | 14 DAYS | 83.9 | 382 | 366 | 0.53 | 0.65 | 0.09 | 0.16 | - | - | - | - | - | - | - | - | - | - | - | - | - | - | - | - | - | - | - | - | - | - |
| D | ISR | EXPD.023 | 3 | 14 DAYS | 82.5 | 371 | 369 | 0.56 | 0.69 | 0.16 | 0.15 | - | - | - | - | - | - | - | - | - | - | - | - | - | - | - | - | - | - | - | - | - | - |
| D | ISR | EXPD.024 | 3 | 14 DAYS | 89.6 | 393 | 384 | 0.56 | 0.66 | 0.16 | 0.15 | - | - | - | - | - | - | - | - | - | - | - | - | - | - | - | - | - | - | - | - | - | - |
| D | ISR | EXPD.025 | 3 | 28 DAYS | 79.5 | 391 | 412 | 0.87 | 0.62 | 0.22 | 0.15 | 2.99 | 2.71 | 3.20 | 6.06 | 8.96 | 11.14 | 13.65 | 14.67 | 15.10 | 15.01 | 14.79 | 18.05 | 16.12 | 22.00 | 34.58 | 46.14 | 52.42 | 58.17 | 60.60 | 61.69 | 62.73 | 62.89 |
| D | ISR | EXPD.026 | 3 | 28 DAYS | 82.1 | 351 | 370 | 0.80 | 0.62 | 0.16 | 0.14 | 5.95 | 5.59 | 8.89 | 14.05 | 18.58 | 21.93 | 24.97 | 25.97 | 26.38 | 26.68 | 26.80 | 19.52 | 18.66 | 25.15 | 37.52 | 46.94 | 51.44 | 55.69 | 57.83 | 59.27 | 59.93 | 59.25 |
| D | ISR | EXPD.027 | 3 | 28 DAYS | 79.6 | 411 | 429 | 0.69 | 0.63 | 0.14 | 0.14 | 8.46 | 7.62 | 10.87 | 17.35 | 24.40 | 27.87 | 30.81 | 32.38 | 33.15 | 33.57 | 33.76 | 19.69 | 18.19 | 23.28 | 35.49 | 50.42 | 58.89 | 65.77 | 69.30 | 71.62 | 72.73 | 72.51 |
| D | ISR | EXPD.028 | 3 | 28 DAYS | 98.7 | 384 | 390 | 0.66 | 0.54 | 0.09 | 0.08 | 1.11 | 1.12 | 0.98 | 1.61 | 2.91 | 5.89 | 8.60 | 10.69 | 11.78 | 12.74 | 14.16 | 18.21 | 16.69 | 23.21 | 35.80 | 50.83 | 61.24 | 69.27 | 72.84 | 74.95 | 75.87 | 76.05 |
| D | ISR | EXPD.029 | 3 | 28 DAYS | 77.1 | 427 | 443 | 0.41 | 0.71 | 0.17 | 0.17 | - | - | - | - | - | - | - | - | - | - | - | - | - | - | - | - | - | - | - | - | - | - |
| D | ISR | EXPD.030 | 3 | 28 DAYS | 87.9 | 399 | 395 | 0.60 | 0.72 | 0.13 | 0.16 | - | - | - | - | - | - | - | - | - | - | - | - | - | - | - | - | - | - | - | - | - | - |
| D | ISR | EXPD.031 | 3 | 28 DAYS | 82.6 | 379 | 381 | 0.35 | 0.63 | 0.12 | 0.13 | - | - | - | - | - | - | - | - | - | - | - | - | - | - | - | - | - | - | - | - | - | - |
| D | ISR | EXPD.032 | 3 | 28 DAYS | 86.6 | 394 | 390 | 0.53 | 0.64 | 0.24 | 0.12 | - | - | - | - | - | - | - | - | - | - | - | - | - | - | - | - | - | - | - | - | - | - |
| D | ISR | EXPD.033 | 3 | 56 DAYS | 92.1 | 365 | 431 | 0.41 | 0.66 | 0.12 | 0.16 | 4.97 | 3.87 | 3.87 | 5.99 | 9.52 | 15.63 | 21.29 | 23.23 | 24.70 | 25.51 | 26.09 | 17.68 | 15.54 | 16.14 | 20.64 | 29.23 | 41.24 | 61.33 | 66.85 | 70.20 | 72.41 | 73.69 |
| D | ISR | EXPD.034 | 3 | 56 DAYS | 78.5 | 385 | 443 | 0.47 | 0.75 | 0.11 | 0.19 | 4.10 | 3.63 | 4.17 | 6.23 | 11.73 | 20.80 | 30.24 | 33.05 | 34.77 | 36.11 | 36.68 | 16.66 | 14.51 | 14.04 | 19.12 | 30.49 | 47.34 | 62.79 | 63.89 | 65.36 | 66.11 | 66.52 |
| D | ISR | EXPD.035 | 3 | 56 DAYS | 95 | 379 | 460 | - | - | - | - | 8.59 | 7.49 | 8.59 | 11.93 | 18.73 | 27.14 | 37.35 | 41.14 | 45.61 | 47.43 | 48.62 | 13.93 | 12.36 | 12.90 | 17.77 | 25.83 | 38.00 | 54.83 | 63.70 | 67.67 | 69.90 | 71.22 |
| D | ISR | EXPD.036 | 3 | 56 DAYS | 78.3 | 375 | 417 | - | - | - | - | 5.15 | 4.57 | 5.72 | 9.82 | 16.04 | 22.07 | 27.12 | 28.81 | 30.17 | 30.95 | 31.35 | 16.35 | 15.35 | 17.36 | 27.53 | 42.38 | 53.97 | 63.69 | 67.71 | 70.14 | 71.56 | 72.44 |
| E | ISR | EXPE.001 | 3 | 14 DAYS | - | 376 | 377 | 0.43 | 0.86 | 0.14 | - | 0.80 | - | 0.72 | - | - | 3.90 | - | - | - | 7.88 | - | 15.20 | - | 12.57 | - | - | 45.60 | - | - | - | 57.06 | - |
| E | ISR | EXPE.002 | 3 | 14 DAYS | - | 370 | 376 | 0.42 | 0.81 | 0.17 | 0.17 | 2.23 | - | 2.87 | - | - | 7.74 | - | - | - | 8.78 | - | 22.58 | - | 37.82 | - | - | 56.09 | - | - | - | 59.28 | - |
| E | ISR | EXPE.003 | 3 | 14 DAYS | - | 380 | 367 | - | - | - | - | - | - | - | - | - | - | - | - | - | - | - | - | - | - | - | - | - | - | - | - | - | - |
| E | ISR | EXPE.004 | 3 | 14 DAYS | - | 380 | 380 | - | - | - | - | - | - | - | - | - | - | - | - | - | - | - | - | - | - | - | - | - | - | - | - | - | - |
| E | ISR | EXPE.005 | 3 | 14 DAYS | - | - | 370 | - | - | - | - | - | - | - | - | - | - | - | - | - | - | - | - | - | - | - | - | - | - | - | - | - | - |
| E | ISR | EXPE.006 | 3 | 14 DAYS | - | - | 381 | - | - | - | - | - | - | - | - | - | - | - | - | - | - | - | - | - | - | - | - | - | - | - | - | - | - |
| E | ISR | EXPE.007 | 3 | 14 DAYS | - | - | 391 | - | - | - | - | - | - | - | - | - | - | - | - | - | - | - | - | - | - | - | - | - | - | - | - | - | - |
| E | ISR | EXPE.008 | 3 | 14 DAYS | - | - | 379 | - | - | - | - | - | - | - | - | - | - | - | - | - | - | - | - | - | - | - | - | - | - | - | - | - | - |
| E | ISR | EXPE.009 | 3 | 14 DAYS | - | - | 380 | - | - | - | - | - | - | - | - | - | - | - | - | - | - | - | - | - | - | - | - | - | - | - | - | - | - |
| E | ISR | EXPE.010 | 3 | 14 DAYS | - | - | 397 | - | - | - | - | - | - | - | - | - | - | - | - | - | - | - | - | - | - | - | - | - | - | - | - | - | - |
| E | ISR | EXPE.011 | 6 | 14 DAYS | - | 361 | 370 | 0.33 | 0.67 | 0.13 | 0.15 | 3.57 | - | 3.49 | - | - | 13.34 | - | - | - | 18.34 | - | 17.92 | - | 20.27 | - | - | 38.19 | - | - | - | 47.43 | - |
| E | ISR | EXPE.012 | 6 | 14 DAYS | - | 373 | 391 | 0.52 | 0.71 | 0.16 | 0.16 | 4.57 | - | 5.07 | - | - | 15.99 | - | - | - | 27.35 | - | 19.26 | - | 23.25 | - | - | 52.10 | - | - | - | 65.29 | - |
| E | ISR | EXPE.013 | 3 | 28 DAYS | - | 366 | 398 | 0.51 | 0.91 | 0.21 | 0.18 | 0.83 | - | 4.82 | - | - | 16.96 | - | - | - | 33.62 | - | 14.53 | - | 18.13 | - | - | 48.41 | - | - | - | 69.54 | - |
| E | ISR | EXPE.014 | 3 | 28 DAYS | - | 360 | 400 | 0.53 | 0.81 | 0.19 | 0.18 | 2.78 | - | 3.15 | - | - | 16.05 | - | - | - | 26.18 | - | 10.35 | - | 10.58 | - | - | 25.88 | - | - | - | 36.60 | - |
| E | ISR | EXPE.015 | 3 | 28 DAYS | - | 431 | 439 | 0.45 | 0.62 | 0.16 | 0.16 | 2.26 | - | 3.21 | - | - | - | - | - | - | 11.89 | - | - | - | - | - | - | - | - | - | - | - | - |
| E | ISR | EXPE.016 | 6 | 28 DAYS | - | 357 | 386 | 0.27 | 0.74 | 0.11 | 0.21 | - | - | - | - | - | - | - | - | - | - | - | - | - | - | - | - | - | - | - | - | - | - |
| E | ISR | EXPE.017 | 6 | 28 DAYS | - | 376 | 422 | 0.51 | 0.88 | 0.21 | 0.20 | 8.03 | - | 8.46 | - | - | 29.57 | - | - | - | 49.91 | - | 16.14 | - | 15.92 | - | - | 41.66 | - | - | - | 48.55 | - |
| E | ISR | EXPE.018 | 6 | 28 DAYS | - | 338 | 381 | 0.49 | 0.67 | 0.19 | 0.14 | - | - | - | - | - | - | - | - | - | - | - | - | - | - | - | - | - | - | - | - | - | - |
| F | USU | EXPF.001 | 1 | 14 DAYS | 116 | 390 | 385 | 0.49 | 0.68 | 0.06 | 0.14 | 3.19 | 1.95 | 2.57 | 5.22 | 8.81 | 11.61 | 14.10 | 17.45 | 19.71 | 20.10 | 20.26 | 15.35 | 15.66 | 17.45 | 18.39 | 21.58 | 26.18 | 36.62 | 45.35 | 50.73 | 52.21 | 53.53 |
| F | USU | EXPF.002 | 1 | 14 DAYS | 70 | 371 | 384 | 0.64 | 0.76 | 0.13 | 0.17 | 5.78 | 5.63 | 8.20 | 13.28 | 20.31 | 24.61 | 29.77 | 33.20 | 35.63 | 36.33 | 37.03 | 15.63 | 14.61 | 17.11 | 26.95 | 40.31 | 48.05 | 54.06 | 57.66 | 60.39 | 61.72 | 61.48 |
| F | USU | EXPF.003 | 1 | 14 DAYS | 73 | 398 | 391 | 0.56 | 0.75 | 0.18 | 0.17 | 5.06 | 4.76 | 5.68 | 8.21 | 10.59 | 14.35 | 20.18 | 23.48 | 23.40 | 27.47 | 26.47 | 7.06 | 7.37 | 14.42 | 30.77 | 41.66 | 46.73 | 51.71 | 54.86 | 56.09 | 55.40 | 54.25 |
| F | USU | EXPF.004 | 1 | 14 DAYS | 73 | 389 | 371 | 0.48 | 0.66 | 0.16 | 0.19 | 9.54 | 8.65 | 10.67 | 13.67 | 17.71 | 22.48 | 23.85 | 26.04 | 27.57 | 28.14 | 28.87 | 16.33 | 18.36 | 25.71 | 40.27 | 47.39 | 51.51 | 56.85 | 58.95 | 60.40 | 60.32 | 59.84 |
| F | USU | EXPF.005 | 2 | 14 DAYS | 99 | 392 | 371 | 0.69 | 0.87 | 0.19 | 0.16 | 3.40 | 0.24 | 4.53 | 7.28 | 10.51 | 13.99 | 17.30 | 19.16 | 20.54 | 20.54 | 17.71 | 14.07 | 14.31 | 19.97 | 27.09 | 34.61 | 39.38 | 44.96 | 56.68 | 59.68 | 59.60 | 59.27 |
| F | USU | EXPF.006 | 2 | 14 DAYS | 97 | 358 | 363 | 0.55 | 0.72 | 0.16 | 0.18 | 0.41 | 0.25 | 3.55 | 6.20 | 11.16 | 16.86 | 22.56 | 25.29 | 26.69 | 27.19 | 27.02 | 12.31 | 12.23 | 20.74 | 31.49 | 39.26 | 45.62 | 51.90 | 54.63 | 55.45 | 55.04 | 53.72 |
| F | USU | EXPF.007 | 1 | 28 DAYS | 76 | 392 | 415 | 0.65 | 0.84 | 0.15 | 0.19 | 6.07 | 5.28 | 7.73 | 13.45 | 22.27 | 28.48 | 37.95 | 43.52 | 46.70 | 48.07 | 48.72 | 14.39 | 12.94 | 17.78 | 27.61 | 39.40 | 46.48 | 51.11 | 54.22 | 55.66 | 57.04 | 57.04 |
| F | USU | EXPF.008 | 1 | 28 DAYS | 70 | 364 | 378 | 0.57 | 0.70 | 0.17 | 0.18 | 5.08 | 4.52 | 6.83 | 11.75 | 21.90 | 19.13 | 35.79 | 42.54 | 47.06 | 48.73 | 49.92 | 11.98 | 10.79 | 15.40 | 21.03 | 25.16 | 30.00 | 40.63 | 45.08 | 48.41 | 49.92 | 51.90 |
| F | USU | EXPF.009 | 1 | 28 DAYS | 95 | 374 | 395 | 0.59 | 0.73 | 0.20 | 0.18 | 2.35 | 2.35 | 3.11 | 5.70 | 10.48 | 15.65 | 20.51 | 22.33 | 23.70 | 23.92 | 23.85 | 11.39 | 11.24 | 17.77 | 30.61 | 41.01 | 47.24 | 53.24 | 55.97 | 58.10 | 59.09 | 59.39 |
| F | USU | EXPF.010 | 2 | 28 DAYS | 91 | 394 | 420 | 0.91 | 0.78 | 0.23 | 0.17 | - | - | - | - | - | - | - | - | - | - | - | - | - | - | - | - | - | - | - | - | - | - |
| F | USU | EXPF.011 | 2 | 28 DAYS | 91 | 398 | 417 | 0.62 | 0.77 | 0.19 | 0.19 | 2.45 | 1.73 | 9.14 | 16.04 | 25.04 | 32.73 | 38.78 | 41.37 | 42.88 | 43.74 | 43.60 | 12.88 | 10.79 | 14.39 | 17.19 | 30.07 | 45.04 | 49.57 | 52.23 | 53.67 | 54.46 | 54.46 |
| F | USU | EXPF.012 | 2 | 28 DAYS | 101 | 373 | 395 | 0.53 | 0.72 | 0.22 | 0.18 | 5.92 | 5.32 | 7.44 | 13.14 | 20.28 | 26.66 | 31.67 | 33.65 | 28.33 | 30.23 | 26.13 | 11.01 | 10.78 | 17.16 | 28.86 | 28.25 | 37.29 | 40.03 | 41.47 | 43.82 | 45.80 | 49.52 |
| F | USU | EXPF.013 | 1 | 56 DAYS | 89 | 378 | 446 | 0.50 | 0.76 | 0.16 | 0.18 | 1.55 | 1.14 | 2.09 | 5.31 | 9.89 | 13.92 | 16.95 | 17.69 | 17.96 | 17.69 | 17.02 | 14.19 | 12.71 | 11.97 | 12.58 | 16.41 | 21.26 | 35.04 | 43.79 | 48.50 | 50.85 | 53.21 |
| F | USU | EXPF.014 | 1 | 56 DAYS | 103 | 345 | 403 | 0.45 | 0.75 | 0.17 | 0.15 | 1.04 | 0.89 | 0.82 | 1.12 | 1.34 | 2.53 | 5.88 | 8.26 | 10.42 | 11.61 | 13.47 | 10.50 | 9.90 | 14.59 | 26.43 | 37.37 | 43.18 | 49.35 | 50.17 | 49.06 | 47.34 | 45.78 |
| F | USU | EXPF.015 | 1 | 56 DAYS | 86 | 350 | 416 | 0.78 | 0.83 | 0.30 | 0.18 | 6.35 | 5.91 | 5.70 | 7.14 | 9.45 | 11.25 | 19.76 | 27.76 | 32.24 | 35.55 | 38.08 | 14.57 | 13.85 | 13.41 | 14.06 | 17.24 | 21.20 | 33.32 | 42.19 | 47.60 | 49.40 | 52.72 |
| F | USU | EXPF.016 | 1 | 56 DAYS | 91 | 360 | 421 | 0.64 | 0.76 | 0.23 | 0.20 | 3.99 | 3.14 | 2.49 | 2.07 | 2.42 | 3.78 | 10.48 | 19.67 | 27.15 | 29.14 | 30.43 | 11.47 | 10.76 | 15.39 | 27.15 | 28.86 | 24.66 | 32.71 | 42.04 | 46.75 | 29.50 | 20.38 |
| F | USU | EXPF.017 | 3 | 56 DAYS | 110 | 291 | 414 | 0.57 | 0.82 | 0.12 | 0.22 | 5.80 | 5.14 | 7.61 | 12.03 | 18.41 | 22.90 | 26.88 | 28.84 | 30.22 | 30.14 | 30.22 | 6.16 | 7.32 | 10.29 | 15.43 | 20.72 | 25.29 | 33.26 | 38.84 | 42.17 | 43.19 | 43.12 |
| F | USU | EXPF.018 | 3 | 56 DAYS | 66 | 211 | 345 | 0.67 | 0.74 | 0.17 | 0.19 | 5.65 | 5.65 | 9.04 | 16.61 | 36.43 | 31.22 | 35.91 | 41.13 | 39.65 | 40.26 | 40.17 | - | - | - | - | - | - | - | - | - | - | - |
| F | USU | EXPF.019 | 3 | 56 DAYS | 80 | 303 | 445 | 0.68 | 0.87 | 0.22 | 0.20 | 4.52 | 4.18 | 3.44 | 3.64 | 4.58 | 6.13 | 10.72 | 19.62 | 29.53 | 33.84 | 36.67 | - | - | - | - | - | - | - | - | - | - | - |
| F | USU | EXPF.020 | 3 | 56 DAYS | 65 | 283 | 420 | 0.71 | 0.67 | 0.17 | 0.16 | - | - | - | - | - | - | - | - | - | - | - | 15.71 | 15.36 | 20.57 | 31.93 | 40.36 | 46.79 | 56.86 | 56.64 | 58.43 | 59.29 | 59.07 |
| G | ISR | EXPG.001 | 10 | 56 DAYS | - | - | 374 | 0.60 | 0.63 | - | - | 11.26 | - | - | - | - | 44.32 | - | - | - | 57.59 | - | 18.76 | - | - | - | - | 58.55 | - | - | - | 73.20 | - |
| G | ISR | EXPG.002 | 10 | 56 DAYS | - | - | 406 | 0.61 | 0.68 | - | - | 11.59 | - | - | - | - | 40.49 | - | - | - | 51.41 | - | 18.83 | - | - | - | - | 55.95 | - | - | - | 69.14 | - |
| G | ISR | EXPG.003 | 10 | 56 DAYS | - | - | 393 | 0.56 | 0.65 | - | - | 12.15 | - | - | - | - | 43.02 | - | - | - | 57.66 | - | 17.72 | - | - | - | - | 54.60 | - | - | - | 67.05 | - |
| G | ISR | EXPG.004 | 10 | 56 DAYS | - | - | 423 | 0.59 | 0.69 | - | - | 7.86 | - | - | - | - | 31.51 | - | - | - | 41.76 | - | 17.50 | - | - | - | - | 51.13 | - | - | - | 64.22 | - |
| G | ISR | EXPG.005 | 10 | 56 DAYS | - | - | 392 | 0.58 | 0.66 | - | - | 9.07 | - | - | - | - | 35.14 | - | - | - | 45.23 | - | 17.50 | - | - | - | - | 51.73 | - | - | - | 65.65 | - |
| G | ISR | EXPG.006 | 10 | 56 DAYS | - | - | 429 | 0.64 | 0.71 | - | - | 9.22 | - | - | - | - | 36.06 | - | - | - | 51.12 | - | 20.13 | - | - | - | - | 56.39 | - | - | - | 68.82 | - |
| G | ISR | EXPG.007 | 10 | 56 DAYS | - | - | 432 | 0.58 | 0.66 | - | - | 12.60 | - | - | - | - | 40.04 | - | - | - | 50.28 | - | 18.34 | - | - | - | - | 49.04 | - | - | - | 59.81 | - |
| G | ISR | EXPG.008 | 10 | 56 DAYS | - | - | 433 | 0.61 | 0.69 | - | - | 7.88 | - | - | - | - | 26.97 | - | - | - | 34.70 | - | 14.41 | - | - | - | - | 53.62 | - | - | - | 69.23 | - |
| H | USU | EXPH.001 | 3 | 03 DAYS | 130.1 | 573 | 570 | 0.99 | 0.95 | 0.26 | 0.22 | - | - | - | - | - | - | - | - | - | - | - | - | - | - | - | - | - | - | - | - | - | - |
| H | USU | EXPH.002 | 3 | 03 DAYS | 127.99 | 548 | 530 | 0.83 | 0.77 | 0.26 | 0.21 | - | - | - | - | - | - | - | - | - | - | - | - | - | - | - | - | - | - | - | - | - | - |
| H | USU | EXPH.003 | 3 | 03 DAYS | 101.3 | 540 | 524 | 1.08 | 0.89 | 0.27 | 0.22 | - | - | - | - | - | - | - | - | - | - | - | - | - | - | - | - | - | - | - | - | - | - |
| H | USU | EXPH.004 | 3 | 03 DAYS | 116.73 | 606 | 571 | 0.91 | 0.82 |  |  | - | - | - | - | - | - | - | - | - | - | - | - | - | - | - | - | - | - | - | - | - | - |
| H | USU | EXPH.005 | 3 | 03 DAYS | 120.84 | 548 | 541 | 0.80 | 0.97 | 0.27 | 0.22 | - | - | - | - | - | - | - | - | - | - | - | - | - | - | - | - | - | - | - | - | - | - |
| H | USU | EXPH.006 | 3 | 07 DAYS | 122 | 483 | 512 | 0.90 | 0.77 | 0.22 | 0.19 | - | - | - | - | - | - | - | - | - | - | - | - | - | - | - | - | - | - | - | - | - | - |
| H | USU | EXPH.007 | 3 | 07 DAYS | 79 | 417 | 412 | 0.63 | 0.83 | 0.21 | 0.20 | - | - | - | - | - | - | - | - | - | - | - | - | - | - | - | - | - | - | - | - | - | - |
| H | USU | EXPH.008 | 3 | 07 DAYS | 101 | 569 | 542 | 0.76 | 0.86 | 0.25 | 0.23 | - | - | - | - | - | - | - | - | - | - | - | - | - | - | - | - | - | - | - | - | - | - |
| H | USU | EXPH.009 | 5 | 07 DAYS | 73 | 428 | 400 | 0.64 | 0.76 | 0.17 | 0.19 | - | - | - | - | - | - | - | - | - | - | - | - | - | - | - | - | - | - | - | - | - | - |
| H | USU | EXPH.010 | 9 | 07 DAYS | 92 | 450 | 438 | 0.86 | 0.81 | 0.14 | 0.19 | - | - | - | - | - | - | - | - | - | - | - | - | - | - | - | - | - | - | - | - | - | - |
| H | USU | EXPH.011 | 3 | 14 DAYS | 88 | 479 | 507 | 0.69 | 0.90 | 0.20 | 0.20 | - | - | - | - | - | - | - | - | - | - | - | - | - | - | - | - | - | - | - | - | - | - |
| H | USU | EXPH.012 | 3 | 14 DAYS | 119 | 605 | 640 | 0.62 | 0.98 | 0.23 | 0.29 | - | - | - | - | - | - | - | - | - | - | - | - | - | - | - | - | - | - | - | - | - | - |
| H | USU | EXPH.013 | 3 | 14 DAYS | 123 | 601 | 685 | 1.00 | 1.24 | 0.21 | 0.34 | - | - | - | - | - | - | - | - | - | - | - | - | - | - | - | - | - | - | - | - | - | - |
| H | USU | EXPH.014 | 3 | 14 DAYS | 123 | 547 | 576 | 0.83 | 0.94 | 0.27 | 0.23 | - | - | - | - | - | - | - | - | - | - | - | - | - | - | - | - | - | - | - | - | - | - |
| H | USU | EXPH.015 | 5 | 14 DAYS | 73 | 448 | 465 | 0.81 | 0.92 | 0.18 | 0.21 | - | - | - | - | - | - | - | - | - | - | - | - | - | - | - | - | - | - | - | - | - | - |
| H | USU | EXPH.016 | 5 | 14 DAYS | 83 | 487 | 487 | 0.61 | 0.89 | 0.12 | 0.24 | - | - | - | - | - | - | - | - | - | - | - | - | - | - | - | - | - | - | - | - | - | - |
| H | USU | EXPH.017 | 9 | 14 DAYS | 128 | 501 | 524 | 0.67 | 0.88 | 0.20 | 0.22 | - | - | - | - | - | - | - | - | - | - | - | - | - | - | - | - | - | - | - | - | - | - |
| H | USU | EXPH.018 | 9 | 14 DAYS | 84 | 451 | 482 | 0.75 | 0.87 | 0.21 | 0.22 | - | - | - | - | - | - | - | - | - | - | - | - | - | - | - | - | - | - | - | - | - | - |
| H | USU | EXPH.019 | 2 | 28 days | 107 | 320 | 363 | - | - | - | - | - | - | - | - | - | - | - | - | - | - | - | - | - | - | - | - | - | - | - | - | - | - |
| H | USU | EXPH.020 | 7 | 28 days | 88 | 333 | 388 | - | - | - | - | - | - | - | - | - | - | - | - | - | - | - | - | - | - | - | - | - | - | - | - | - | - |
| H | USU | EXPH.021 | 7 | 28 days | 75 | 313 | 353 | - | - | - | - | - | - | - | - | - | - | - | - | - | - | - | - | - | - | - | - | - | - | - | - | - | - |
| H | USU | EXPH.022 | 3 | 90 DAYS | 99 | 429 | 579 | 1.11 | 1.13 | 0.35 | 0.27 | - | - | - | - | - | - | - | - | - | - | - | - | - | - | - | - | - | - | - | - | - | - |
| H | USU | EXPH.023 | 3 | 90 DAYS | 107 | 485 | 650 | 1.03 | 1.11 | 0.28 | 0.27 | - | - | - | - | - | - | - | - | - | - | - | - | - | - | - | - | - | - | - | - | - | - |
| H | USU | EXPH.024 | 5 | 90 DAYS | 73 | 465 | 579 | 1.04 | 1.06 | 0.30 | 0.26 | - | - | - | - | - | - | - | - | - | - | - | - | - | - | - | - | - | - | - | - | - | - |
| H | USU | EXPH.025 | 5 | 90 DAYS | 114 | 435 | 539 | 1.03 | 1.14 | 0.21 | 0.28 | - | - | - | - | - | - | - | - | - | - | - | - | - | - | - | - | - | - | - | - | - | - |
| H | USU | EXPH.026 | 5 | 90 DAYS | 82 | 373 | 448 | 0.84 | 0.94 | 0.25 | 0.22 | - | - | - | - | - | - | - | - | - | - | - | - | - | - | - | - | - | - | - | - | - | - |
| H | USU | EXPH.027 | 9 | 90 DAYS | 107 | 451 | 557 | 0.90 | 1.04 | 0.23 | 0.28 | - | - | - | - | - | - | - | - | - | - | - | - | - | - | - | - | - | - | - | - | - | - |
| H | USU | EXPH.028 | 9 | 90 DAYS | 85 | 428 | 504 | 0.83 | 0.99 | 0.27 | 0.23 | - | - | - | - | - | - | - | - | - | - | - | - | - | - | - | - | - | - | - | - | - | - |
| H | USU | EXPH.029 | 9 | 90 DAYS | 84 | 491 | 553 | 1.06 | 0.95 | 0.29 | 0.26 | - | - | - | - | - | - | - | - | - | - | - | - | - | - | - | - | - | - | - | - | - | - |
| I | UMN | EXPI.001 | 4 | 00 Days | 69 | 340 | 340 | 0.57 | 0.60 | 0.08 | 0.12 | - | - | - | - | - | - | - | - | - | - | - | - | - | - | - | - | - | - | - | - | - | - |
| I | UMN | EXPI.002 | 4 | 00 Days | 94 | 375 | 375 | 0.53 | 0.62 | 0.17 | 0.15 | - | - | - | - | - | - | - | - | - | - | - | - | - | - | - | - | - | - | - | - | - | - |
| I | UMN | EXPI.003 | 4 | 00 Days | 74 | 360 | 360 | 0.60 | 0.66 | 0.20 | 0.14 | - | - | - | - | - | - | - | - | - | - | - | - | - | - | - | - | - | - | - | - | - | - |
| I | UMN | EXPI.004 | 4 | 00 Days | 84 | 369 | 369 | 0.55 | 0.64 | 0.16 | 0.16 | - | - | - | - | - | - | - | - | - | - | - | - | - | - | - | - | - | - | - | - | - | - |
| I | UMN | EXPI.005 | 4 | 00 Days | 118 | 354 | 354 | 0.55 | 0.63 | 0.16 | 0.14 | - | - | - | - | - | - | - | - | - | - | - | - | - | - | - | - | - | - | - | - | - | - |
| I | UMN | EXPI.006 | 4 | 00 Days | 68 | 360 | 360 | 0.58 | 0.60 | 0.16 | 0.15 | - | - | - | - | - | - | - | - | - | - | - | - | - | - | - | - | - | - | - | - | - | - |
| I | UMN | EXPI.007 | 4 | 00 Days | 91 | 344 | 344 | 0.53 | 0.59 | 0.14 | 0.14 | - | - | - | - | - | - | - | - | - | - | - | - | - | - | - | - | - | - | - | - | - | - |
| I | UMN | EXPI.008 | 4 | 00 Days | 86 | 352 | 352 | 0.55 | 0.61 | 0.14 | 0.14 | - | - | - | - | - | - | - | - | - | - | - | - | - | - | - | - | - | - | - | - | - | - |
| I | UMN | EXPI.009 | 4 | 03 DAYS | 89 | 406 | 388 | 0.65 | 0.69 | 0.15 | 0.17 | - | - | - | - | - | - | - | - | - | - | - | - | - | - | - | - | - | - | - | - | - | - |
| I | UMN | EXPI.010 | 4 | 03 DAYS | 104 | 430 | 407 | 0.69 | 0.63 | 0.16 | 0.15 | - | - | - | - | - | - | - | - | - | - | - | - | - | - | - | - | - | - | - | - | - | - |
| I | UMN | EXPI.011 | 4 | 03 DAYS | 87 | 390 | 377 | 0.65 | 0.68 | 0.16 | 0.16 | - | - | - | - | - | - | - | - | - | - | - | - | - | - | - | - | - | - | - | - | - | - |
| I | UMN | EXPI.012 | 4 | 03 DAYS | 97 | 392 | 375 | 0.63 | 0.62 | 0.17 | 0.14 | - | - | - | - | - | - | - | - | - | - | - | - | - | - | - | - | - | - | - | - | - | - |
| I | UMN | EXPI.013 | 4 | 03 DAYS | 74 | 363 | 352 | 0.65 | 0.61 | 0.15 | 0.14 | - | - | - | - | - | - | - | - | - | - | - | - | - | - | - | - | - | - | - | - | - | - |
| I | UMN | EXPI.014 | 4 | 03 DAYS | 97 | 372 | 362 | 0.61 | 0.65 | 0.16 | 0.14 | - | - | - | - | - | - | - | - | - | - | - | - | - | - | - | - | - | - | - | - | - | - |
| I | UMN | EXPI.015 | 4 | 03 DAYS | 99 | 354 | 343 | 0.81 | 0.64 | 0.16 | 0.15 | - | - | - | - | - | - | - | - | - | - | - | - | - | - | - | - | - | - | - | - | - | - |
| I | UMN | EXPI.016 | 4 | 03 DAYS | 117 | 360 | 346 | 0.83 | 0.59 | 0.12 | 0.14 | - | - | - | - | - | - | - | - | - | - | - | - | - | - | - | - | - | - | - | - | - | - |
| I | UMN | EXPI.017 | 4 | 07 DAYS | 79 | 376 | 376 | 0.55 | 0.64 | 0.12 | 0.17 | - | - | - | - | - | - | - | - | - | - | - | - | - | - | - | - | - | - | - | - | - | - |
| I | UMN | EXPI.018 | 4 | 07 DAYS | 78 | 390 | 396 | 0.76 | 0.75 | 0.16 | 0.18 | - | - | - | - | - | - | - | - | - | - | - | - | - | - | - | - | - | - | - | - | - | - |
| I | UMN | EXPI.019 | 4 | 07 DAYS | 75 | 388 | 379 | 0.69 | 0.71 | 0.14 | 0.15 | - | - | - | - | - | - | - | - | - | - | - | - | - | - | - | - | - | - | - | - | - | - |
| I | UMN | EXPI.020 | 4 | 07 DAYS | 82 | 382 | 365 | 0.63 | 0.00 | 0.16 | 0.15 | - | - | - | - | - | - | - | - | - | - | - | - | - | - | - | - | - | - | - | - | - | - |
| I | UMN | EXPI.021 | 4 | 07 DAYS | 69 | 342 | 335 | 0.50 | 0.58 | 0.14 | 0.14 | - | - | - | - | - | - | - | - | - | - | - | - | - | - | - | - | - | - | - | - | - | - |
| I | UMN | EXPI.022 | 4 | 07 DAYS | 87 | 356 | 343 | 0.61 | 0.55 | 0.13 | 0.14 | - | - | - | - | - | - | - | - | - | - | - | - | - | - | - | - | - | - | - | - | - | - |
| I | UMN | EXPI.023 | 4 | 07 DAYS | 94 | 405 | 387 | 0.58 | 0.64 | 0.17 | 0.15 | - | - | - | - | - | - | - | - | - | - | - | - | - | - | - | - | - | - | - | - | - | - |
| I | UMN | EXPI.024 | 4 | 14 DAYS | 79 | 397 | 416 | 0.67 | 0.77 | 0.20 | 0.20 | - | - | - | - | - | - | - | - | - | - | - | - | - | - | - | - | - | - | - | - | - | - |
| I | UMN | EXPI.025 | 4 | 14 DAYS | 88 | 365 | 372 | 0.57 | 0.72 | 0.14 | 0.17 | - | - | - | - | - | - | - | - | - | - | - | - | - | - | - | - | - | - | - | - | - | - |
| I | UMN | EXPI.026 | 4 | 14 DAYS | 74 | 380 | 379 | 0.57 | 0.70 | 0.18 | 0.15 | - | - | - | - | - | - | - | - | - | - | - | - | - | - | - | - | - | - | - | - | - | - |
| I | UMN | EXPI.027 | 4 | 14 DAYS | 60 | 364 | 362 | 0.57 | 0.69 | 0.14 | 0.15 | - | - | - | - | - | - | - | - | - | - | - | - | - | - | - | - | - | - | - | - | - | - |
| I | UMN | EXPI.028 | 4 | 14 DAYS | 75 | 379 | 383 | 0.59 | 0.73 | 0.17 | 0.16 | - | - | - | - | - | - | - | - | - | - | - | - | - | - | - | - | - | - | - | - | - | - |
| I | UMN | EXPI.029 | 4 | 14 DAYS | 77 | 380 | 376 | 0.59 | 0.68 | 0.16 | 0.16 | - | - | - | - | - | - | - | - | - | - | - | - | - | - | - | - | - | - | - | - | - | - |
| I | UMN | EXPI.030 | 4 | 14 DAYS | 82 | 358 | 351 | 0.54 | 0.69 | 0.16 | 0.16 | - | - | - | - | - | - | - | - | - | - | - | - | - | - | - | - | - | - | - | - | - | - |
| I | UMN | EXPI.031 | 4 | 14 DAYS | 67 | 372 | 371 | 0.57 | 0.66 | 0.16 | 0.16 | - | - | - | - | - | - | - | - | - | - | - | - | - | - | - | - | - | - | - | - | - | - |
| I | UMN | EXPI.032 | 4 | 21 DAYS | 94 | 386 | 393 | 0.59 | 0.76 | 0.17 | 0.18 | - | - | - | - | - | - | - | - | - | - | - | - | - | - | - | - | - | - | - | - | - | - |
| I | UMN | EXPI.033 | 4 | 21 DAYS | 80 | 365 | 378 | 0.57 | 0.70 | 0.15 | 0.16 | - | - | - | - | - | - | - | - | - | - | - | - | - | - | - | - | - | - | - | - | - | - |
| I | UMN | EXPI.034 | 4 | 21 DAYS | 104 | 384 | 390 | 0.57 | 0.75 | 0.11 | 0.16 | - | - | - | - | - | - | - | - | - | - | - | - | - | - | - | - | - | - | - | - | - | - |
| I | UMN | EXPI.035 | 4 | 21 DAYS | 93 | 372 | 380 | 0.75 | 0.71 | 0.17 | 0.18 | - | - | - | - | - | - | - | - | - | - | - | - | - | - | - | - | - | - | - | - | - | - |
| I | UMN | EXPI.036 | 4 | 21 DAYS | 102 | 418 | 434 | 0.63 | 0.69 | 0.19 | 0.16 | - | - | - | - | - | - | - | - | - | - | - | - | - | - | - | - | - | - | - | - | - | - |
| I | UMN | EXPI.037 | 4 | 21 DAYS | 100 | 393 | 402 | 0.55 | 0.68 | 0.17 | 0.15 | - | - | - | - | - | - | - | - | - | - | - | - | - | - | - | - | - | - | - | - | - | - |
| I | UMN | EXPI.038 | 4 | 21 DAYS | 100 | 412 | 442 | 0.55 | 0.73 | 0.20 | 0.18 | - | - | - | - | - | - | - | - | - | - | - | - | - | - | - | - | - | - | - | - | - | - |
| I | UMN | EXPI.039 | 4 | 21 DAYS | 105 | 428 | 436 | 0.60 | 0.73 | 0.21 | 0.17 | - | - | - | - | - | - | - | - | - | - | - | - | - | - | - | - | - | - | - | - | - | - |
| I | UMN | EXPI.040 | 4 | 48 DAYS | 92 | 380 | 437 | 0.54 | 0.71 | 0.18 | 0.17 | - | - | - | - | - | - | - | - | - | - | - | - | - | - | - | - | - | - | - | - | - | - |
| I | UMN | EXPI.041 | 4 | 48 DAYS | 81 | 383 | 458 | 0.57 | 0.72 | 0.19 | 0.18 | - | - | - | - | - | - | - | - | - | - | - | - | - | - | - | - | - | - | - | - | - | - |
| I | UMN | EXPI.042 | 4 | 48 DAYS | 66 | 375 | 434 | 0.56 | 0.72 | 0.19 | 0.18 | - | - | - | - | - | - | - | - | - | - | - | - | - | - | - | - | - | - | - | - | - | - |
| I | UMN | EXPI.043 | 4 | 48 DAYS | 89 | 372 | 438 | 0.73 | 0.77 | 0.21 | 0.19 | - | - | - | - | - | - | - | - | - | - | - | - | - | - | - | - | - | - | - | - | - | - |
| I | UMN | EXPI.044 | 4 | 48 DAYS | 78 | 384 | 450 | 0.53 | 0.74 | 0.18 | 0.18 | - | - | - | - | - | - | - | - | - | - | - | - | - | - | - | - | - | - | - | - | - | - |
| I | UMN | EXPI.045 | 4 | 48 DAYS | 90 | 411 | 466 | 0.72 | 0.76 | 0.19 | 0.00 | - | - | - | - | - | - | - | - | - | - | - | - | - | - | - | - | - | - | - | - | - | - |
| I | UMN | EXPI.046 | 4 | 48 DAYS | 75 | 392 | 444 | 0.62 | 0.73 | 0.19 | 0.17 | - | - | - | - | - | - | - | - | - | - | - | - | - | - | - | - | - | - | - | - | - | - |
| I | UMN | EXPI.047 | 4 | 48 DAYS | 76 | 388 | 430 | 0.55 | 0.70 | 0.19 | 0.17 | - | - | - | - | - | - | - | - | - | - | - | - | - | - | - | - | - | - | - | - | - | - |
| J | UMN | EXPJ.001 | 8 | 03 DAYS | 106 | 399 | 382 | 0.79 | 0.60 | 0.14 | 0.16 | - | - | - | - | - | - | - | - | - | - | - | - | - | - | - | - | - | - | - | - | - | - |
| J | UMN | EXPJ.002 | 8 | 03 DAYS | 79 | 396 | 381 | 0.65 | 0.60 | 0.14 | 0.16 | - | - | - | - | - | - | - | - | - | - | - | - | - | - | - | - | - | - | - | - | - | - |
| J | UMN | EXPJ.003 | 8 | 03 DAYS | 74 | 406 | 385 | 0.63 | 0.63 | 0.18 | 0.15 | - | - | - | - | - | - | - | - | - | - | - | - | - | - | - | - | - | - | - | - | - | - |
| J | UMN | EXPJ.004 | 8 | 03 DAYS | 78 | 373 | 360 | 0.58 | 0.60 | 0.15 | 0.14 | - | - | - | - | - | - | - | - | - | - | - | - | - | - | - | - | - | - | - | - | - | - |
| J | UMN | EXPJ.005 | 8 | 03 DAYS | 75 | 337 | 335 | 0.64 | 0.61 | 0.16 | 0.16 | - | - | - | - | - | - | - | - | - | - | - | - | - | - | - | - | - | - | - | - | - | - |
| J | UMN | EXPJ.006 | 8 | 03 DAYS | 90 | 330 | 326 | 0.60 | 0.59 | 0.14 | 0.16 | - | - | - | - | - | - | - | - | - | - | - | - | - | - | - | - | - | - | - | - | - | - |
| J | UMN | EXPJ.007 | 8 | 03 DAYS | 89 | 350 | 348 | 0.70 | 0.62 | 0.17 | 0.14 | - | - | - | - | - | - | - | - | - | - | - | - | - | - | - | - | - | - | - | - | - | - |
| J | UMN | EXPJ.008 | 8 | 03 DAYS | 92 | 338 | 339 | 0.56 | 0.63 | 0.16 | 0.17 | - | - | - | - | - | - | - | - | - | - | - | - | - | - | - | - | - | - | - | - | - | - |
| J | UMN | EXPJ.009 | 8 | 07 DAYS | 74 | 370 | 358 | 0.63 | 0.57 | 0.16 | 0.14 | - | - | - | - | - | - | - | - | - | - | - | - | - | - | - | - | - | - | - | - | - | - |
| J | UMN | EXPJ.010 | 8 | 07 DAYS | 93 | 387 | 375 | 0.54 | 0.62 | 0.15 | 0.15 | - | - | - | - | - | - | - | - | - | - | - | - | - | - | - | - | - | - | - | - | - | - |
| J | UMN | EXPJ.011 | 8 | 07 DAYS | 100 | 392 | 374 | 0.52 | 0.58 | 0.15 | 0.15 | - | - | - | - | - | - | - | - | - | - | - | - | - | - | - | - | - | - | - | - | - | - |
| J | UMN | EXPJ.012 | 8 | 07 DAYS | 94 | 393 | 387 | 0.57 | 0.62 | 0.14 | 0.14 | - | - | - | - | - | - | - | - | - | - | - | - | - | - | - | - | - | - | - | - | - | - |
| J | UMN | EXPJ.013 | 8 | 07 DAYS | 75 | 346 | 337 | 0.48 | 0.48 | 0.14 | 0.13 | - | - | - | - | - | - | - | - | - | - | - | - | - | - | - | - | - | - | - | - | - | - |
| J | UMN | EXPJ.014 | 8 | 07 DAYS | 88 | 318 | 317 | 0.44 | 0.53 | 0.14 | 0.14 | - | - | - | - | - | - | - | - | - | - | - | - | - | - | - | - | - | - | - | - | - | - |
| J | UMN | EXPJ.015 | 8 | 07 DAYS | 68 | 365 | 356 | 0.50 | 0.61 | 0.14 | 0.14 | - | - | - | - | - | - | - | - | - | - | - | - | - | - | - | - | - | - | - | - | - | - |
| J | UMN | EXPJ.016 | 8 | 07 DAYS | 81 | 356 | 349 | 0.60 | 0.61 | 0.15 | 0.14 | - | - | - | - | - | - | - | - | - | - | - | - | - | - | - | - | - | - | - | - | - | - |
| J | UMN | EXPJ.017 | 8 | 14 DAYS | 74 | 387 | 390 | 0.54 | 0.67 | 0.15 | 0.14 | - | - | - | - | - | - | - | - | - | - | - | - | - | - | - | - | - | - | - | - | - | - |
| J | UMN | EXPJ.018 | 8 | 14 DAYS | 109 | 396 | 401 | 0.51 | 0.66 | 0.14 | 0.16 | - | - | - | - | - | - | - | - | - | - | - | - | - | - | - | - | - | - | - | - | - | - |
| J | UMN | EXPJ.019 | 8 | 14 DAYS | 89 | 360 | 358 | 0.36 | 0.57 | 0.15 | 0.15 | - | - | - | - | - | - | - | - | - | - | - | - | - | - | - | - | - | - | - | - | - | - |
| J | UMN | EXPJ.020 | 8 | 14 DAYS | 62 | 352 | 355 | 0.40 | 0.59 | 0.13 | 0.13 | - | - | - | - | - | - | - | - | - | - | - | - | - | - | - | - | - | - | - | - | - | - |
| J | UMN | EXPJ.021 | 8 | 14 DAYS | 92 | 345 | 353 | 0.46 | 0.62 | 0.17 | 0.15 | - | - | - | - | - | - | - | - | - | - | - | - | - | - | - | - | - | - | - | - | - | - |
| J | UMN | EXPJ.022 | 8 | 14 DAYS | 100 | 344 | 369 | 0.63 | 0.63 | 0.16 | 0.15 | - | - | - | - | - | - | - | - | - | - | - | - | - | - | - | - | - | - | - | - | - | - |
| J | UMN | EXPJ.023 | 8 | 14 DAYS | 79 | 322 | 344 | 0.49 | 0.69 | 0.12 | 0.15 | - | - | - | - | - | - | - | - | - | - | - | - | - | - | - | - | - | - | - | - | - | - |
| J | UMN | EXPJ.024 | 8 | 14 DAYS | 79 | 323 | 340 | 0.52 | 0.60 | 0.15 | 0.14 | - | - | - | - | - | - | - | - | - | - | - | - | - | - | - | - | - | - | - | - | - | - |
| J | UMN | EXPJ.025 | 8 | 21 DAYS | 60 | 359 | 372 | 0.58 | 0.65 | 0.15 | 0.14 | - | - | - | - | - | - | - | - | - | - | - | - | - | - | - | - | - | - | - | - | - | - |
| J | UMN | EXPJ.026 | 8 | 21 DAYS | 58 | 375 | 384 | 0.58 | 0.67 | 0.14 | 0.15 | - | - | - | - | - | - | - | - | - | - | - | - | - | - | - | - | - | - | - | - | - | - |
| J | UMN | EXPJ.027 | 8 | 21 DAYS | 96 | 354 | 356 | 0.38 | 0.59 | 0.13 | 0.13 | - | - | - | - | - | - | - | - | - | - | - | - | - | - | - | - | - | - | - | - | - | - |
| J | UMN | EXPJ.028 | 8 | 21 DAYS | 91 | 377 | 388 | 0.43 | 0.66 | 0.13 | 0.14 | - | - | - | - | - | - | - | - | - | - | - | - | - | - | - | - | - | - | - | - | - | - |
| J | UMN | EXPJ.029 | 8 | 21 DAYS | 100 | 342 | 372 | 0.44 | 0.65 | 0.12 | 0.14 | - | - | - | - | - | - | - | - | - | - | - | - | - | - | - | - | - | - | - | - | - | - |
| J | UMN | EXPJ.030 | 8 | 21 DAYS | 100 | 340 | 381 | 0.64 | 0.69 | 0.15 | 0.16 | - | - | - | - | - | - | - | - | - | - | - | - | - | - | - | - | - | - | - | - | - | - |
| J | UMN | EXPJ.031 | 8 | 21 DAYS | 97 | 377 | 388 | 0.51 | 0.66 | 0.15 | 0.15 | - | - | - | - | - | - | - | - | - | - | - | - | - | - | - | - | - | - | - | - | - | - |
| J | UMN | EXPJ.032 | 8 | 21 DAYS | 103 | 354 | 369 | 0.54 | 0.66 | 0.13 | 0.15 | - | - | - | - | - | - | - | - | - | - | - | - | - | - | - | - | - | - | - | - | - | - |
| J | UMN | EXPJ.033 | 8 | 48 DAYS | 78 | 389 | 467 | 0.76 | 0.71 | 0.16 | 0.17 | - | - | - | - | - | - | - | - | - | - | - | - | - | - | - | - | - | - | - | - | - | - |
| J | UMN | EXPJ.034 | 8 | 48 DAYS | 100 | 395 | 458 | 0.36 | 0.73 | 0.21 | 0.17 | - | - | - | - | - | - | - | - | - | - | - | - | - | - | - | - | - | - | - | - | - | - |
| J | UMN | EXPJ.035 | 8 | 48 DAYS | 96 | 372 | 415 | 0.44 | 0.71 | 0.16 | 0.15 | - | - | - | - | - | - | - | - | - | - | - | - | - | - | - | - | - | - | - | - | - | - |
| J | UMN | EXPJ.036 | 8 | 48 DAYS | 84 | 394 | 455 | 0.62 | 0.71 | 0.16 | 0.17 | - | - | - | - | - | - | - | - | - | - | - | - | - | - | - | - | - | - | - | - | - | - |
| J | UMN | EXPJ.037 | 8 | 48 DAYS | 89 | 363 | 459 | 0.72 | 0.72 | 0.21 | 0.18 | - | - | - | - | - | - | - | - | - | - | - | - | - | - | - | - | - | - | - | - | - | - |
| J | UMN | EXPJ.038 | 8 | 48 DAYS | 84 | 340 | 455 | 0.64 | 0.67 | 0.20 | 0.18 | - | - | - | - | - | - | - | - | - | - | - | - | - | - | - | - | - | - | - | - | - | - |
| J | UMN | EXPJ.039 | 8 | 48 DAYS | 81 | 327 | 396 | 0.58 | 0.64 | 0.15 | 0.16 | - | - | - | - | - | - | - | - | - | - | - | - | - | - | - | - | - | - | - | - | - | - |
| J | UMN | EXPJ.040 | 8 | 48 DAYS | 86 | 342 | 429 | 0.60 | 0.64 | 0.17 | 0.15 | - | - | - | - | - | - | - | - | - | - | - | - | - | - | - | - | - | - | - | - | - | - |
| K | UMN | EXPK.001 | 8 | 48 DAYS | 93 | 339 | 402 | 0.63 | 0.66 | 0.20 | 0.17 | 3.18 | 2.98 | 5.59 | 11.57 | - | 24.22 | 26.33 | 27.86 | 28.40 | 26.14 | - | - | - | - | - | - | - | - | - | - | - | - |
| K | UMN | EXPK.002 | 8 | 48 DAYS | 78 | 349 | 428 | 0.59 | 0.69 | 0.20 | 0.16 | 2.60 | 3.30 | 5.76 | 13.46 | - | 30.87 | 36.96 | 38.83 | 39.52 | 39.11 | - | - | - | - | - | - | - | - | - | - | - | - |
| K | UMN | EXPK.003 | 8 | 48 DAYS | 80 | 329 | 389 | 0.58 | 0.69 | 0.16 | 0.14 | 0.72 | 1.31 | 2.70 | 3.36 | - | 15.60 | 21.71 | 24.18 | 26.35 | 28.03 | - | - | - | - | - | - | - | - | - | - | - | - |
| K | UMN | EXPK.004 | 8 | 48 DAYS | 82 | 326 | 394 | 0.55 | 0.72 | 0.16 | 0.15 | 4.59 | 5.31 | 8.45 | 17.25 | - | 36.97 | 40.31 | 40.59 | 41.08 | 40.78 | - | - | - | - | - | - | - | - | - | - | - | - |
| K | UMN | EXPK.005 | 8 | 48 DAYS | 85 | 347 | 436 | 0.64 | 0.69 | 0.19 | 0.17 | 2.33 | 4.05 | 7.13 | 18.20 | - | 30.24 | 30.19 | 40.09 | 42.51 | 42.88 | - | - | - | - | - | - | - | - | - | - | - | - |
| K | UMN | EXPK.006 | 8 | 48 DAYS | 100 | 340 | 419 | 0.43 | 0.72 | 0.16 | 0.17 | 1.98 | 2.28 | 3.44 | 10.85 | - | 21.79 | 25.99 | 26.23 | 27.63 | 27.52 | - | - | - | - | - | - | - | - | - | - | - | - |
| K | UMN | EXPK.007 | 8 | 48 DAYS | 75 | 360 | 439 | 0.53 | 0.67 | 0.15 | 0.17 | 3.14 | 4.05 | 7.58 | 16.60 | - | 34.76 | 40.77 | 42.76 | 43.99 | 44.53 | - | - | - | - | - | - | - | - | - | - | - | - |
| K | UMN | EXPK.008 | 8 | 48 DAYS | 78 | 345 | 439 | 0.65 | 0.66 | 0.19 | 0.17 | 3.92 | 5.89 | 10.14 | 20.56 | - | 40.74 | 47.45 | 49.69 | 50.14 | 51.06 | - | - | - | - | - | - | - | - | - | - | - | - |
| L | USU | EXPL.001 | 1 | 7 days | 93 | 404 | 392 | 0.43 | 0.62 | 0.16 | 0.15 | - | - | - | - | - | - | - | - | - | - | - | - | - | - | - | - | - | - | - | - | - | - |
| L | USU | EXPL.002 | 1 | 7 days | 95 | 342 | 340 | 0.40 | 0.58 | 0.12 | 0.14 | - | - | - | - | - | - | - | - | - | - | - | - | - | - | - | - | - | - | - | - | - | - |
| L | USU | EXPL.003 | 1 | 7 days | 95 | 359 | 362 | 0.42 | 0.56 | 0.14 | 0.16 | - | - | - | - | - | - | - | - | - | - | - | - | - | - | - | - | - | - | - | - | - | - |
| L | USU | EXPL.004 | 1 | 7 days | 91 | 309 | 308 | 0.54 | 0.56 | 0.07 | 0.14 | - | - | - | - | - | - | - | - | - | - | - | - | - | - | - | - | - | - | - | - | - | - |
| L | USU | EXPL.005 | 1 | 7 days | 80 | 318 | 340 | 0.47 | 0.55 | 0.14 | 0.13 | - | - | - | - | - | - | - | - | - | - | - | - | - | - | - | - | - | - | - | - | - | - |
| L | USU | EXPL.006 | 1 | 7 days | 82 | 324 | 356 | 0.47 | 0.59 | 0.13 | 0.14 | - | - | - | - | - | - | - | - | - | - | - | - | - | - | - | - | - | - | - | - | - | - |
| L | USU | EXPL.007 | 1 | 7 days | 89 | 350 | 330 | 0.47 | 0.58 | 0.12 | 0.13 | - | - | - | - | - | - | - | - | - | - | - | - | - | - | - | - | - | - | - | - | - | - |
| L | USU | EXPL.008 | 7 | 7 days | 106 | 323 | 322 | 0.37 | 0.52 | 0.15 | 0.12 | - | - | - | - | - | - | - | - | - | - | - | - | - | - | - | - | - | - | - | - | - | - |
| L | USU | EXPL.009 | 7 | 7 days | 99 | 353 | 359 | 0.52 | 0.63 | 0.16 | 0.16 | - | - | - | - | - | - | - | - | - | - | - | - | - | - | - | - | - | - | - | - | - | - |
| L | USU | EXPL.010 | 7 | 7 days | 90 | 330 | 347 | 0.53 | 0.60 | 0.15 | 0.15 | - | - | - | - | - | - | - | - | - | - | - | - | - | - | - | - | - | - | - | - | - | - |
