# Supplementary material for: Retrospective characterization of a rat model of volumetric muscle loss
Source: BMC Musculoskelet Disord. 2022 Aug 26;23:814. doi: 10.1186/s12891-022-05760-5 (PMC9414143; doi:10.1186/s12891-022-05760-5)
Supplement: Supplementary file 2 — Additional file 2: Table S1. Studies utilizing the 6 mm biopsy punch model of VML in the rat tibialis anterior muscle [9, 11, 14–27]. Table S2. Published studies included for comparative analysis using partial thickness models of VML in the rat TA muscle. Table S3. Best fit parameters of sigmoidal fit for torque-frequency relationship of TA muscles with VML. [file 12891_2022_5760_MOESM2_ESM.docx]

| **Table S1**: Studies Utilizing the 6 mm Biopsy Punch Model of VML in the Rat Tibialis Anterior Muscle | | | | | | |
| --- | --- | --- | --- | --- | --- | --- |
| Study | Experiment Code | Strain | Sex | Institution | Reference | Exclusion  Code |
| Goldman et al. 2017 | A | Lewis | M | USAISR | (14) |  |
| Corona et al. 2018 | B | Lewis | M | USAISR | (10) |  |
| Goldman et al. 2017 | C | Lewis | M | USAISR | (15) |  |
| Aguilar et al. 2018 | D | Lewis | M | USAISR | (8) |  |
| Goldman et al. 2020 | E | Lewis | M | USAISR | (16) |  |
| Dearth et al. | F | Lewis | M | USUHS | Unpublished Data |  |
| Ward et al. 2015 | G | Lewis | M | USAISR | (17) |  |
| Dearth et al. | H | SD | M | USUHS | Unpublished Data |  |
| Sorensen et al 2021 | I | Lewis | M | University of Minnesota | (18) |  |
| Greising et al | J | Lewis | M | University of Minnesota | Unpublished Data |  |
| Greising et al | K | Lewis | M | University of Minnesota | Unpublished Data |  |
| Goldman et al. | L | Lewis | M | USUHS | Unpublished Data |  |
| Garg et al. 2014 | - | Lewis | M | USAISR | (19) | §§§§ |
| Goldman et al. 2017 | - | Lewis | M | USAISR | (20) | § |
| Goldman et al. 2020 | - | SD Dawley | M | USUHS | (21) | § |
| Goldman et al. 2021 | - | SD | M | USUHS | (22) | § |
| Hoffman et al. 2020 | - | Lewis | M | University of Minnesota | (23) | §§§§§ |
| Pilia et al. 2014 | - | Lewis | M | USAISR | (24) | §§ |
| Haas et al 2021 | - | Lewis | M | Saint Louis University | (25) | §§ |
| **Notes**: SD: Sprague Dawley, USAISR: US Army Institute of Surgical Research, USUHS: Uniformed Services University of the Health Sciences. Exclusion codes: § - No unrepaired experimental group, §§ - Bilateral study design, §§§ - No functional data, §§§§ - Disparate muscle function testing methods, §§§§§ - Alternate analysis of already included data | | | | | | |

| **Table S2**: Published studies Included for Comparative Analysis using Partial Thickness Models of VML in the Rat TA Muscle | | | | | |
| --- | --- | --- | --- | --- | --- |
| Publication | Strain | Sex | Surgical Weight | Defect Specifications | Endpoint |
| Wu et al. 2012(26) | Lewis | M | ~350g | 10 × 7 × 3 mm | 16 Weeks |
| Corona et al. 2013(7) | Lewis | M | 325-350g | 10 × 7 × 3 mm | 8 & 16 Weeks |
| Garg et al. 2014(27) | Lewis | M | 426.4±1.7g | 10 × 7 × 3 mm | 8 Weeks |
| Kim et al. 2016(28) | Fischer 344 | M | 312 ± 3 g | 8mm Ø × 3mm | 8 & 12 Weeks |
| Passipieri et al. 2017(29) | Lewis | F | 203.4 ± 0.8 g | ~20% of TA | 8 & 12 Weeks |

| **Table S3:** Best Fit Parameters of Sigmoidal Fit for Torque-Frequency Relationship of TA Muscles with VML | | | | |
| --- | --- | --- | --- | --- |
| **Study Endpoint** | **Parameters** | | | |
|  | **Top** | **Bottom** | **Hill Slope** | **LogEC50** |
| Day 03 | 8.47 ± 1.82 | 0.64 ± 0.62 | 4.41 ± 6.24 | 92.83 ± 5.29 |
| Day 07 | 21.85 ± 0.46 | 3.54 ± 0.31 | 3.99 ± 0.83 | 63.55 ± 0.57 |
| Day 14 | 27.46 ± 1.14 | 3.62 ± 1.66 | 8.08 ± 7.06 | 59.44 ± 1.51 |
| Day 21 | 35.57 ± 2.36 | 6.90 ± 1.91 | 4.08 ± 3.30 | 55.15 ± 1.67 |
| Day 28 | 31.85 ± 2.78 | 4.04 ± 2.15 | 3.21 ± 2.66 | 52.18 ± 2.07 |
| Day 48 | 37.77 ± 2.19 | 3.08 ± 2.00 | 3.91 ± 2.39 | 46.93 ± 1.36 |
| Day 56 | 35.26 ± 1.45 | 5.63 ± 0.94 | 2.78 ± 0.52 | 58.04 ± 1.26 |
| Day 90 | 43.08 ± 2.10 | 5.48 ± 2.14 | 4.28 ± 2.47 | 53.98 ± 1.26 |
| ANOVA | P<0.001 | P=0.021 | P=0.157 | P<0.001 |

# 
